# Perspectives on mitochondrial relevance in cardiac ischemia/reperfusion injury

**DOI:** 10.3389/fcell.2022.1082095

**Published:** 2022-12-06

**Authors:** Gaia Pedriali, Daniela Ramaccini, Esmaa Bouhamida, Mariusz R. Wieckowski, Carlotta Giorgi, Elena Tremoli, Paolo Pinton

**Affiliations:** ^1^ Maria Cecilia Hospital, GVM Care and Research, Cotignola, Italy; ^2^ Laboratory of Mitochondrial Biology and Metabolism, Nencki Institute of Experimental Biology, Warsaw, Poland; ^3^ Laboratory for Technologies of Advanced Therapies (LTTA), Department of Medical Science, Section of Experimental Medicine, University of Ferrara, Ferrara, Italy

**Keywords:** mitochondria, cardiac ischemia/reperfusion, mitophagy, mitochondrial dynamics, mitochondrial biogenesis, PTP

## Abstract

Cardiovascular disease is the most common cause of death worldwide and in particular, ischemic heart disease holds the most considerable position. Even if it has been deeply studied, myocardial ischemia-reperfusion injury (IRI) is still a side-effect of the clinical treatment for several heart diseases: ischemia process itself leads to temporary damage to heart tissue and obviously the recovery of blood flow is promptly required even if it worsens the ischemic injury. There is no doubt that mitochondria play a key role in pathogenesis of IRI: dysfunctions of these important organelles alter cell homeostasis and survival. It has been demonstrated that during IRI the system of mitochondrial quality control undergoes alterations with the disruption of the complex balance between the processes of mitochondrial fusion, fission, biogenesis and mitophagy. The fundamental role of mitochondria is carried out thanks to the finely regulated connection to other organelles such as plasma membrane, endoplasmic reticulum and nucleus, therefore impairments of these inter-organelle communications exacerbate IRI. This review pointed to enhance the importance of the mitochondrial network in the pathogenesis of IRI with the aim to focus on potential mitochondria-targeting therapies as new approach to control heart tissue damage after ischemia and reperfusion process.

## Introduction

As an aerobic muscular organ, the myocardium needs to be perfused with blood rich in oxygen, provided by the coronary circulation, for metabolism and contraction. Ischemic heart disease (IHD) occurs as a consequence of the imbalance between the demand and the supply of oxygenated blood to cardiac tissue; adult cardiac myocytes, in the condition of reduced oxygen, become incapable of maintaining intracellular metabolism, leading to cell apoptosis and necrosis and serious tissue damage. It should be noted that tissue injury strictly depends on ischemic duration, thus a major effort should be given to reduce the time of intervention and to assure a rapid onset of the reperfusion. Reperfusion, however, aggravates the damage, leading to activation of myocardial cell damage eventually resulting in uncontrolled cell death: this event is usually known as ischemia-reperfusion injury (IRI) and has substantial effects on the clinical outcome of the patient, especially because adult cardiac tissue is almost unable to regenerate after injury, resulting in a permanent damage (Araszkiewicz et al., 2013; Frank et al., 2012).

According to World Health Organization (WHO), cardiovascular diseases (CVDs) are the leading causes of death, IHD is the second global cause of disability-adjusted life years and the first global cause of death with an impact of 8,9 million of deaths in the world per year (https://www.who.int/data/gho/data/themes/mortality-and-global-health-estimates). Global Burden of Disease study estimated 197 million prevalent cases of IHD worldwide, causing 49,2% of total CVDs deaths (Roth et al., 2020). IHD is not only a disease of the old people in rich countries, but also several studies indicate an impact of this pathological condition on working-age adults and increasing trouble in low- and middle-income countries (Murray and Lopez, 1997; Abegunde et al., 2007). A positive fact is that the mortality due to IHD has declined over the past 4 decades in western countries thanks to significant progress in interventional procedures and medical treatments, to the reduction of risk factors and to lifestyle improvements.

As clinical definition, myocardial infarction (ST-segment elevation myocardial infarction (STEMI) and non-STEMI), stable angina and ischemic cardiomyopathy are part of the group of IHD (Heusch, 2019).

In fact, this type of disease is complex and multifactorial: several studies have linked IHD to environmental factors such as physical activity, stress, diet, smoking and to a variety of cardiovascular risk factors, as family history of cardiovascular disease, hypercholesterolemia, hypertension, or diabetes (Khera et al., 2016; Whayne and Saha, 2019). A not-modifiable risk factor is gender: there is a higher prevalence of IHD in men in comparison to women, in a way independent by age between the two genders but with an earlier age of onset in men (Khan et al., 2020).

Due to the impact of IHD on global health, its pathophysiology has been deeply studied and several efforts have been made to depict molecular pathways underlying its complexity in order to ameliorate clinical interventions and therapies. At present, there are not effective therapies in use focusing on reduction of IRI, thus the understanding of the molecular mechanisms underlying this unavoidable damage is fundamental in order to develop cardioprotective strategies. In particular, taking into account that mitochondria have an important role in IHD pathophysiology, recent efforts oriented toward the study of these organelles with the aim of ameliorating clinical approaches to treat IHD. It should be noted that, IRI is not a specific cardiac process, but it occurs in several other organs leading to a variety of diseases. For instance, IRI is an important complication after kidney and heart transplantation (Salvadori et al., 2015; Liu et al., 2018), leading to primary graft failure and reducing the survival of recipients. IRI has also been found as a common feature of ischemic stroke (L et al., 2016). Thus studies concerning IRI focused on the disentangling of the molecular mechanisms involved in its onset are still important in order to develop new therapies for this pathological process.

Cardiac tissue requires a large amount of energy to carry out its function, in particular for contractile capacity and for the control of electrical conduction (Murphy et al., 2016) and these processes are supported by the intensified mitochondrial network of cardiomyocytes. In fact, in cardiac myocytes mitochondria occupy almost 30% of the total volume. To note, in mammalian cells, mitochondria are primarily localized around the nucleus; on the contrary, in adult cardiomyocytes, mitochondria are highly organized and localized in three different areas among the cardiac fibers that can be evidenced with multimodal microscopy (Barzda et al., 2005). This highly coordinated localization is reached during the development, in fact, fetal mitochondria are not well organized, they lack well-formed cristae and of a specific localization (Garbern and Lee, 2021). The different localization of mitochondria in cardiomyocytes may be associated to different functions: subsarcolemmal (SSM), which has been suggested to supply ATP for the active transport of metabolites and electrolytes across the sarcolemma; perinuclear (PNM), with a not yet clear function but with a potential relation to nuclear metabolism; and intermyofibrillar mitochondria (IFM), which are the most active for oxidative metabolism and connected to muscle contraction (Shimada et al., 1984; Hollander et al., 2014).

**FIGURE 1:**
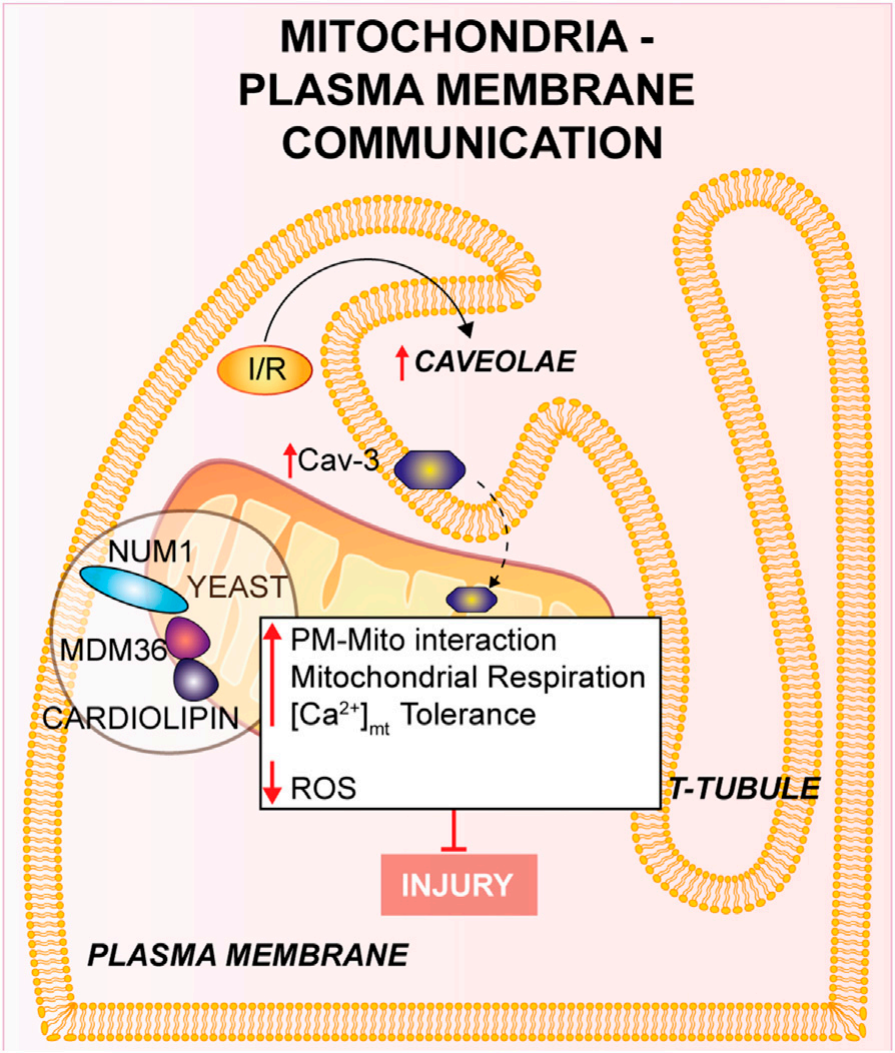
Mitochondria-Plasma membrane communication. Num1/Mdm36 complex anchors the PM to the OMM, with the involvement of cardiolipin. Cardiomyocyte’s T-tubules displays mitochondria-associated caveolae (PM invaginations), which number is increased in response to I/R. Furthermore, the overexpression of Cav-3 showed a cardioprotective effect against I/R.

**FIGURE 2:**
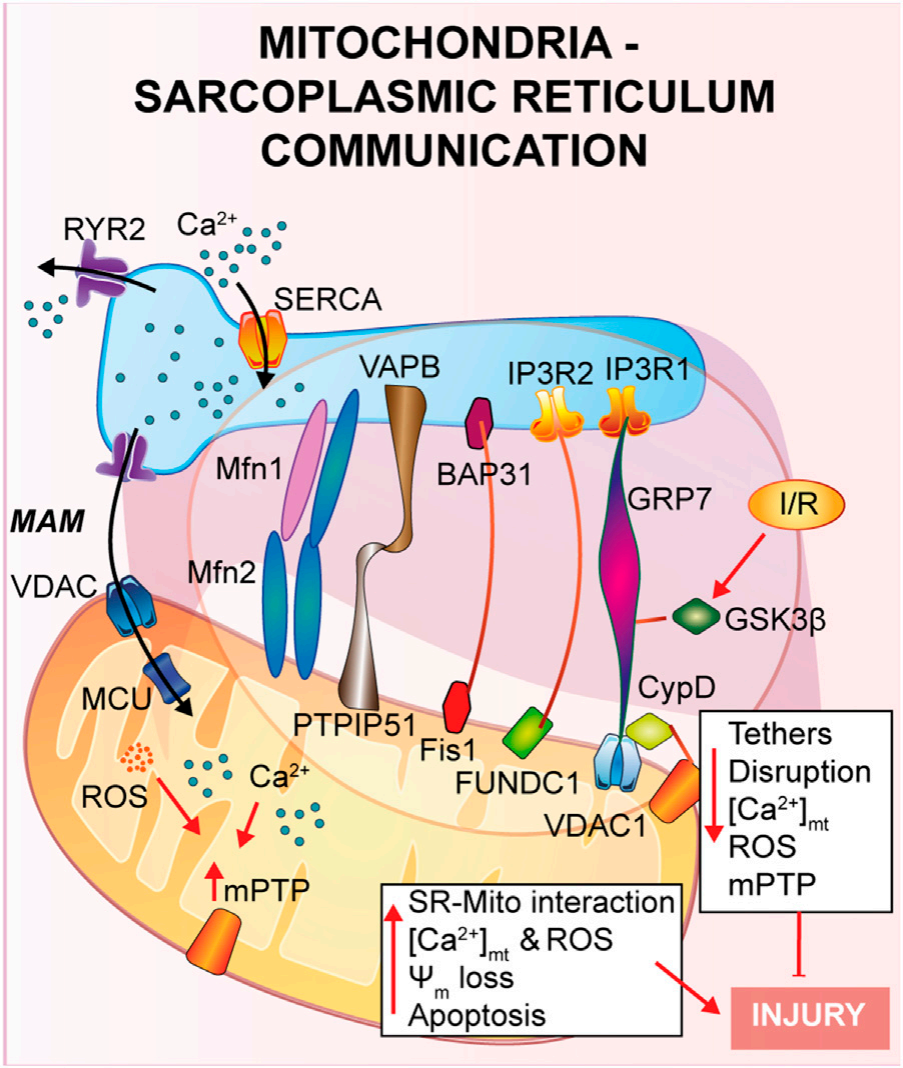
Mitochondria-Sarcoplasmic reticulum interaction. This type of interaction forms a region called MAM, fundamental for the presence of several proteins that guarantee a correct tethering and regulate Ca^2+^ cycle. In general, a reduction in the SR-mitochondria tethering correlates with decrease in mitochondrial Ca^2+^ uptake, reduction of cell death and cardioprotection to IRI.

**FIGURE 3:**
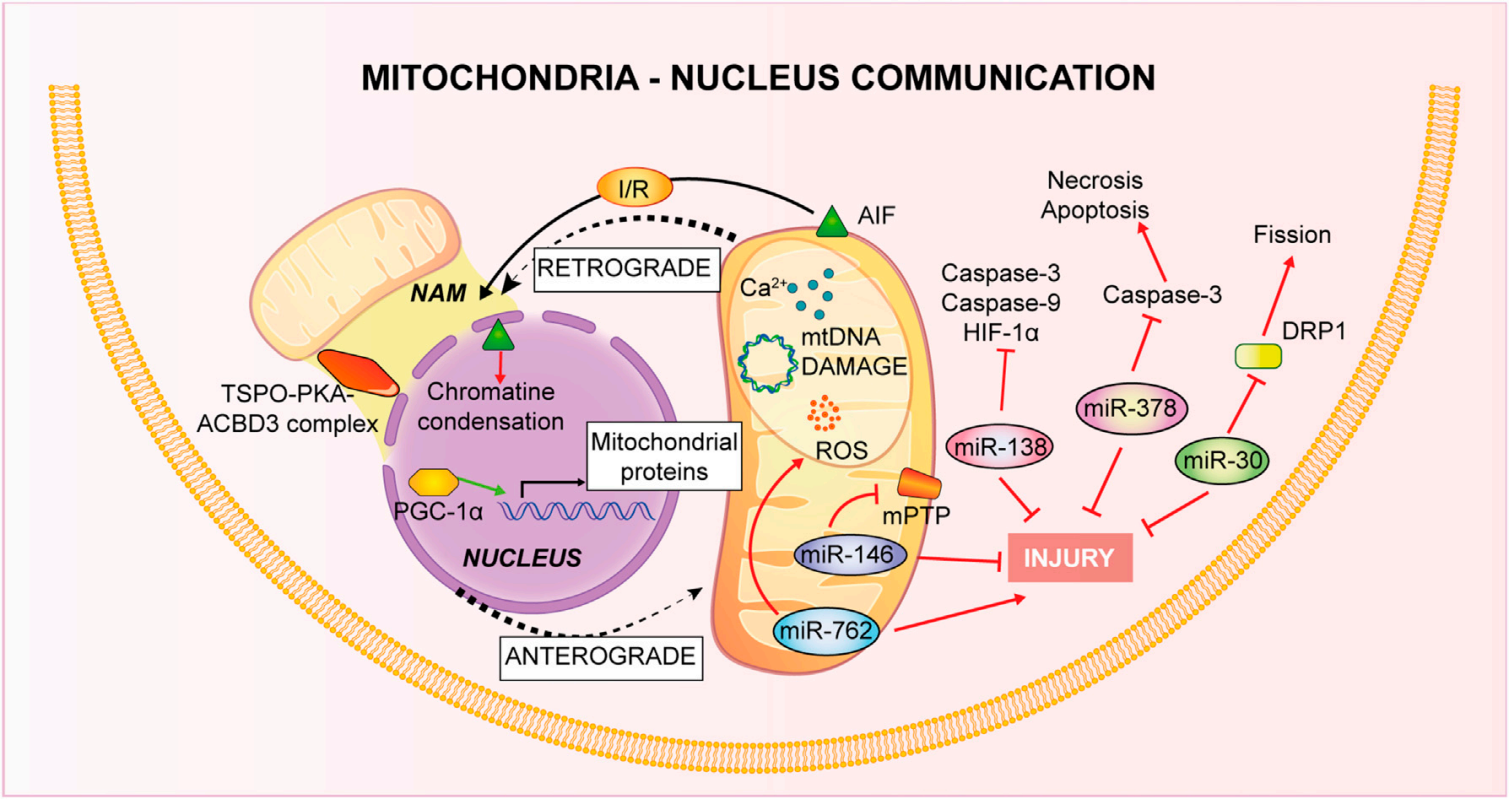
Mitochondria-Nucleus communication. Nucleus control of mitochondrial health is named anterograde signaling, through nuclear genes encoding mitochondrial proteins and *via* miRNAs. Mitochondria, conversely, use a retrograde signaling induced by Ca^2+^-dependent stress, mtDNA damage, metabolic disorders and loss of ψ_m_.

Mitochondria are semi-autonomous organelles, highly motile and able to integrate and communicate with different compartments: in the last years, the direct contact between the mitochondria and other intracellular organelles has been deeply investigated, particularly with the endoplasmic reticulum (ER) (Angebault et al., 2020; Marchi et al., 2014). The mitochondria and ER contacts are among the most critical studied contact sites, probably because their occurrence is considered as a hub for Ca^2+^ homeostasis and the exchange of lipid membrane (Lee and Min, 2018). Nevertheless, communication between the mitochondria and other intracellular organelles has been observed in several studies, albeit the nature of all the contacts is still unclear (for details see Figures 1–3).

**FIGURE 4:**
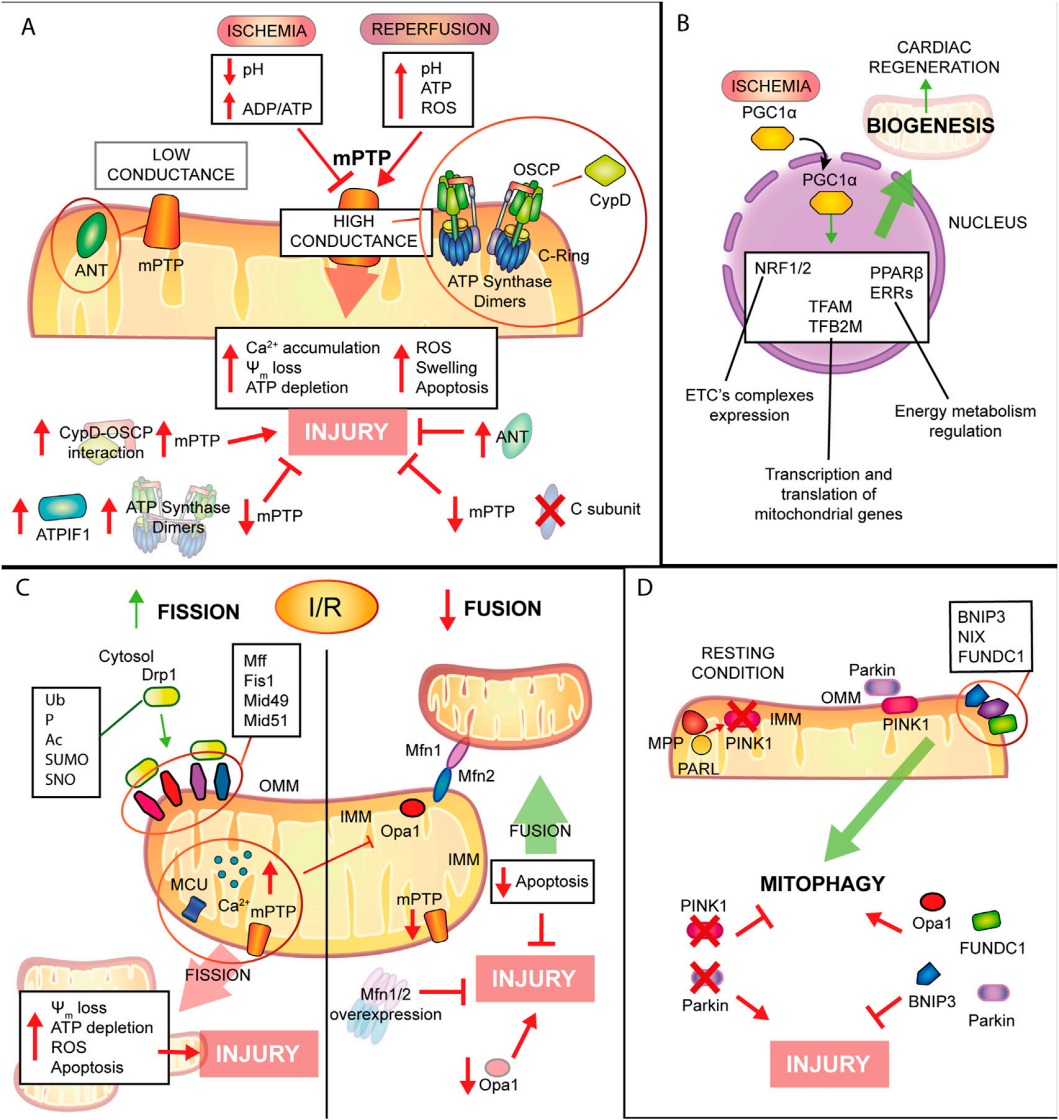
Mitochondrial remodelling and state of health in I/R condition. **(A)** mPTP involvement in IRI. Schematic representation of the main molecular events involving mPTP in IRI. During ischemia mPTP opening is inhibited, after reperfusion this multiprotein complex opening triggers mitochondrial reactions which lead to cardiomyocytes death. **(B)**. Mitochondrial biogenesis. The biogenic process is activated during ischemia with positive consequences in order to improve cellular metabolism and energy production and to promote cardiac regeneration. **(C)**. Mitochondrial dynamics. Fission process is enhanced during I/R leading to tissue injury. Contrariwise, mitochondrial fusion is reduced and increase of this process showed beneficial effects against IRI. **(D)**. Mitophagic process. Induction of mitophagy during myocardial infarction has cardioprotective effects on the condition that is not a chronic activation, very harmful for cardiac tissue.

Mitochondria’s plethora of roles includes ATP production through oxidative phosphorylation (OXPHOS), reactive oxygen species (ROS) production, programmed cell death control, intracellular Ca^2+^ homeostasis (Galluzzi et al., 2012). Thus, taken into consideration their importance, it is obvious that mitochondrial dysfunction is linked to the pathogenesis of different cardiovascular disease (Bonora et al., 2019; Pedriali et al., 2020). The mitochondrial involvement is of greatest interest especially for IHD. During ischemia the lack of oxygen stops the mitochondrial respiratory chain and the consequent ATP production (Hausenloy and Yellon, 2013), it leads to an intracellular Ca^2+^ overload that, at the moment of reperfusion, provokes Ca^2+^ buffering by mitochondria, increases oxidative stress, mitochondrial permeability pore (mPTP) opening, which in turn contributes to cardiomyocytes death (Morciano et al., 2015). Beyond inter-organelle interactions, mitochondria state of health is controlled by their morphology, with an extremely precise interchange of process of fusion and fission or biogenesis and mitophagy (as described in Figure 4).

**FIGURE 5:**
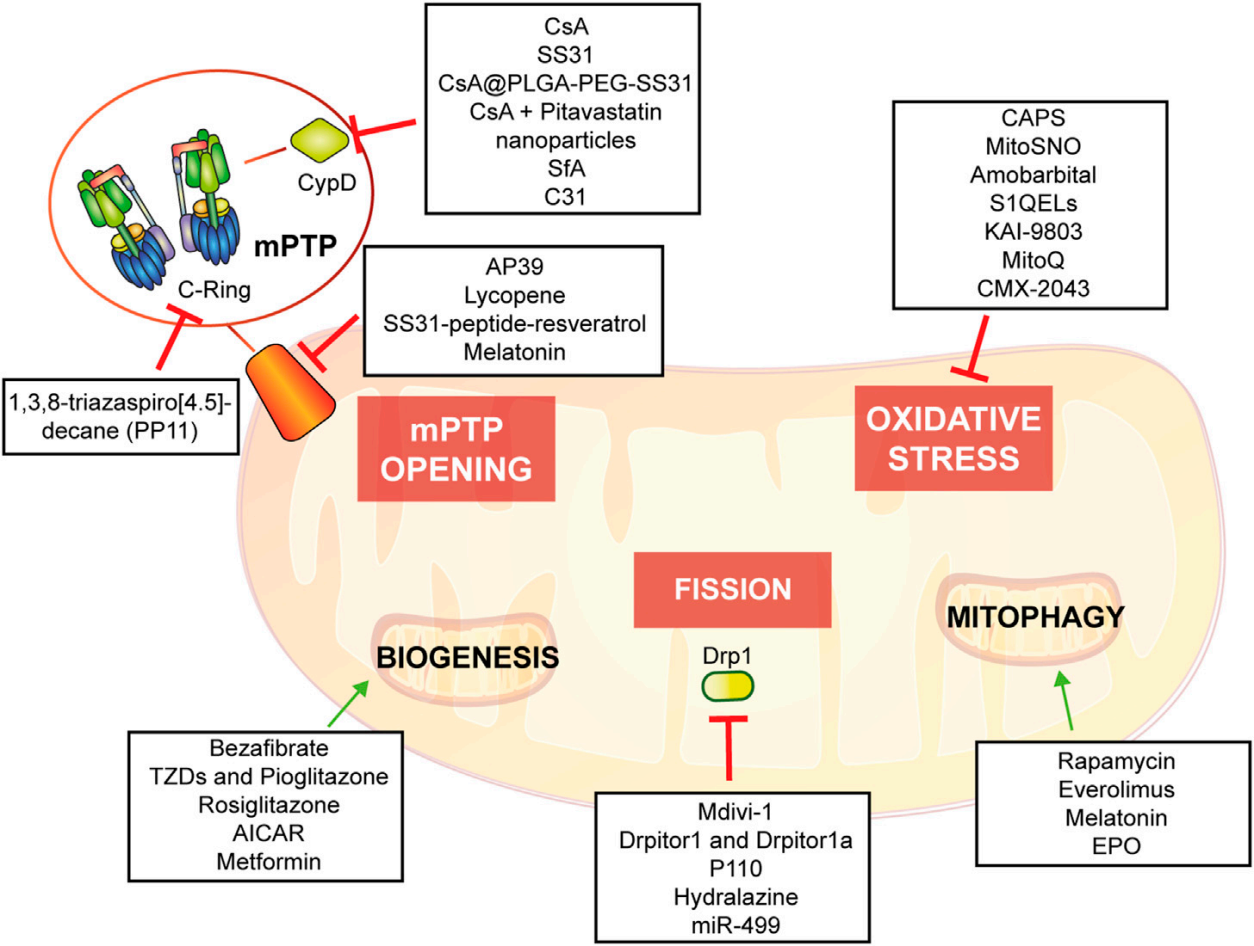
Potential mitochondria-targeting therapied as new approach to control heart tissue damage.

The objective of this review is to describe the relevance of mitochondria in molecular and cellular mechanisms underlying IRI and to describe up-to-date evidence of mitochondria-based potential therapies providing an in-depth understanding of possible therapeutic intervention (Figure 5).

## Inter-organelle communication in I/R

### Mitochondria-endoplasmic reticulum

A physical and functional crosstalk between mitochondria and other organelles, such as plasma membrane (PM), nucleus and sarcoplasmic reticulum (SR) has been previously reported. SR-mitochondria interaction, called mitochondria-associate ER/SR membranes (MAMs), is the first and the most described contact, and it involves several proteins that guarantee a correct tethering and communication between the two organelles (Pinton, 2018; Missiroli et al., 2017; Marchi et al., 2017; Pedriali et al., 2017). In cardiac muscle, SR-mitochondrial contact is a critical regulator of Ca^2+^ cycle during excitation-contraction coupling (ECC), a process that requires a huge amount of ATP supplied by mitochondria during every heartbeat (Denton and McCormack, 1990; Bers, 2008; Cao et al., 2019). Briefly, upon action potential, Ca^2+^ enters from the extracellular space into the cytosol through L-Type Ca^2+^ channel located on the PM, stimulating a large Ca^2+^ release from the SR *via* type 2 ryanodine receptor (RyR2), in a calcium-induced calcium released (CICR) manner, ultimately promoting cardiomyocyte contraction. Activation of the sarcoendoplasmic reticulum Ca^2+^ ATPase (SERCA) brings back Ca^2+^ into the SR, the main intracellular Ca^2+^ store, together with Na^+^/Ca^2+^ exchanger (NCX) on the PM, which clears out Ca^2+^ from the cytosol, promoting cardiomyocyte relaxation. The ECC occurs at the dyadic clefts, the space between the SR and T-tubule membranes, but mitochondria are excluded from this zone (Sharma et al., 2000). Thus, it is still of interest to answer the question whether and how mitochondria contribute to buffering Ca^2+^ for energy production on a beat-to-beat basis (O'Rourke and Blatter, 2009; Fabiato, 1983; De la Fuente and Sheu, 2019).

As already described in the previous section, once Ca^2+^ is released by the SR *via* RyR2, it diffuses into the mitochondria intermembrane space *via* voltage-dependent anion channel 2 (VDAC2) and then is taken up into the mitochondrial matrix *via* mitochondrial calcium uniporter complex (MCUcx) (Giorgi et al., 2018). It has been reported that MCUcx is an ion channel with low affinity for calcium but highly selective (Kirichok et al., 2004). Thus, in order to make possible the mitochondrial Ca^2+^ uptake, Ca^2+^ transfer between SR-mitochondria occurs at the microdomain level (Ramesh et al., 1998; Sharma et al., 2000) where the ions channels assigned for SR-Ca^2+^ release and mitochondria-Ca^2+^ uptake are closely connected. Early studies using transmission electron microscopy and electron tomography (Boncompagni et al., 2009; Hayashi et al., 2009), have shown the existence of these microdomains, but the proteins involved and the molecular structure of these tethers are still under investigation (Filadi et al., 2018). Mitofusin 2 (Mfn2) is the first protein identified to be part of the tethers between ER and mitochondria, however contrasting findings regarding its role have been reported. As it will be described, Mfn2 is a mitochondrial GTPase protein involved in mitochondrial fusion, which has been found to localized also at the ER/SR, where it can form homo- or heterodimers with either Mfn2 or Mfn1. Chen et al. showed that mitofusin 1 (Mfn1) deletion, during the embryonic stage, reveals no differences in distances and Ca^2+^ levels in both SR and mitochondria (Chen et al., 2012). On the contrary, cardiomyocytes specific Mfn2 gene deletion induces a “dissociation” between the two organelles, leading to a Ca^2+^ rise in SR, which is accompanied by a reduction in mitochondrial Ca^2+^ uptake, and a lower mPTP activation during reperfusion (Papanicolaou et al., 2011), thus protecting the myocardium from IRI.

In agreement with the previous study, Hall et al. performed a cardiac-specific deletion of both Mfn1 and Mfn2, leading to the disruption of the tethering SR-mitochondria, attenuating ROS burst and mitochondrial Ca^2+^ overload and showing that hearts deficient in both Mfn1 and Mfn2 are less sensitive to IRI (Hall et al., 2016).

Another complex involved in the SR-mitochondria tethering is the VDAC1/GRP75/IP3R1 channel complex. The mitochondrial stress 70 protein, also known as GRP75, is the bridge between the IP3R1, on the ER side, and VDAC1 at the outer mitochondrial membrane (OMM) (Perrone et al., 2020). Interestingly, during hypoxia and reoxygenation, endothelial cells exhibit an increase in the interaction among the proteins of the VDAC1/GRP75/IP3R1 complex, promoting mitochondrial Ca^2+^ overload and subsequent cell death (He et al., 2015). Moreover, Paillard et al., identified CypD, a Ca^2+^ sensitive mitochondrial chaperone known as a regulator of mPTP opening, as a new player in the VDAC1/GRP75/IP3R1 complex (Paillard et al., 2013). These authors performed experiments on cardiomyoblasts and cardiomyocytes and, through independent genetic deletion and pharmacological inhibition of each component of the complex under hypoxia-reoxygenation condition, cardiac cells showed a reduction in the mitochondrial Ca^2+^ uptake and a reduction of the mPTP opening that correlates with disruption of the SR-mitochondria tethers and Ca^2+^ signaling (Paillard et al., 2013). In addition, these authors obtained the same results upon deletion of Mfn2, confirming its key role in maintaining the correct mitochondrial-SR Ca^2+^ crosstalk, fundamental for cellular response to IRI (Paillard et al., 2013).

In a more recent study, also glycogen synthase kinase 3β (GSK3β), a serine/threonine-protein kinase for glycogen synthase, has been identified as a regulator of the VDAC1/GRP75/IP3R1 complex (Gomez et al., 2016). It was already reported that phosphorylation of GSK3β reduces mPTP opening mediating cardioprotection (Nishihara et al., 2007; Juhaszova et al., 2004; Gomez et al., 2008; Tong et al., 2002), but the mechanism of action was still unknown. The authors proposed that upon ischemia and reperfusion (I/R), GSK3β localizes at the MAMs and physically interacts with each protein of the Ca^2+^-channel complex (VDAC1/GRP75/IP3R1/CypD) and not with RyRs, suggesting a new role as a structural protein of the complex (Gomez et al., 2016). Accordingly, pharmacological inhibition of GSK3β promotes myocardial protection by reducing mitochondrial Ca^2+^ uptake, which is due to disruption of the interaction of IP3R with the other proteins forming the complex, and not due to the binding of GSK3β on IP3R, which remains unchanged (Gomez et al., 2016).

Another less studied protein is the mitochondrial protein tyrosine phosphatase interacting protein 51 (PTPIP51) which is known to interact with the ER vesicle-associated membrane protein-associated protein B (VAPB) forming a bridge between the two organelles and regulating Ca^2+^ homeostasis (De Vos et al., 2012). Qiao et al. showed that PTPIP51 is upregulated upon I/R, leading to cardiomyocytes cell death (Qiao et al., 2017). During myocardial infarction, PTPIP51 promotes mitochondria-SR contact, therefore enhancing Ca^2+^ transfer from SR to mitochondria.

The mitophagic protein fun14 domain-containing protein 1 (FUNDC1) is known to localize at the OMM where it interacts with microtubule-associated protein 1A/1B-light chain 3 (LC3) for autophagosome recruitment; interestingly, it has been recently shown a key role of FUNDC1 as an ER-mitochondrial tethers (Wu et al., 2017; Wu et al., 2016). During hypoxia FUNDC1 interacts with calnexin, an ER protein, and promotes mitochondrial fission by recruiting dynamin-related peptide 1 (Drp1) (Wu et al., 2016). Moreover, another group reported that FUNDC1 modulates ER-Ca^2+^ release by binding IP3R2. Indeed, ablation of FUNDC1 induces ER-mitochondrial dissociation, decreasing mitochondrial Ca^2+^ uptake, promoting mitochondrial fission and cardiac dysfunction upon ischemic stress (Wu et al., 2017).

Other two proteins, known as regulators of apoptosis, were identified in the ER-mitochondrial communication: the mitochondrial fission protein 1 (Fis1) and B cell receptor activators (BAP31) at the ER membrane (Iwasawa et al., 2011). During hypoxia-reoxygenation Fis1 stimulates SR-Ca^2+^ release through interaction with BAP31, which, in turn, promotes Bax and Bak translocation to the mitochondria and the initiation of apoptosis (Lu et al., 2010). In agreement with these studies, a recent article by Cheng et al., has shown that loss of the Fis-BAP31 crosstalk protects hearts from myocardial infarction by downregulating mitochondrial ROS production and c-Jun N-terminal kinase (JNK) pathway activation, thus inhibiting cardiomyocyte cell death (Cheng et al., 2021).

All together, these data highlight the importance of the correct interaction between SR-mitochondria for the maintaining a correct Ca^2+^ homeostasis: a reduction in the ER-mitochondria tethering correlates with a decrease in mitochondrial Ca^2+^ uptake, reducing cardiac myocytes cell death, thus providing a novel platform for preventing IRI.

### Mitochondria-plasma membrane

The contact between the mitochondria and the PM has been disregarded and, to our knowledge, only few insights delineated mitochondria-PM relationships and their molecular elements. This contact might have an extensive impact on mitochondrial function and cell physiology, but results of these studies are still limited and the molecular bases of this crosstalk are still unclear. Multiple observational studies over the past 50 years have proposed a specific attachment between the mitochondria and PM in neuronal and epithelial cells using electron microscopy, in which the mitochondria-PM contact has been found in close proximity (Fridolfsson et al., 2012; Montes de Oca Balderas, 2021).

The mitochondria-PM interaction has been poorly described at the molecular level in yeast (Westermann, 2015). During yeast cell division mitochondria exhibit a tether, termed the mitochondria-ER cortex anchor, with the PM which is mainly mediated through nuclear migration 1 (Num1) and mitochondrial distribution and morphology 36 (Mdm36). Num1 plays an essential role in the mitochondria-PM interaction in which it anchors directly with the OMM to PM by its pleckstrin homology domain in PM, as well as to the mitochondrial network through the coiled-coil domain, where cardiolipin, in addition to other proteins negatively charged located to the cristae junction, is mainly involved (Ping et al., 2016). Moreover, Num1 forms a complex with Mdm36 and their localization is on the cortical part of the mitochondrial tubules (Lackner et al., 2013); it has been shown that this complex has significant effects on mitochondrial inheritance and dynamics *via* the mitochondria-PM tethering (Lawrence and Mandato, 2013). Num1/Mdm36 complex plays an essential role in the mitochondrial morphology and both proteins are considered as key factors of a mitochondria-PM tether complex (Westermann, 2015). Therefore, it has been suggested that other proteins participate to the mitochondria-PM contact beyond the complex Num1/Mdm36 (Lackner et al., 2013), even if more in-depth analyses are needed. Further research should focus on the identification of new proteins orchestrating these processes and their effect on the contact sites.

In cardiomyocytes from multiple species, one piece of evidence has identified a close cohesion of mitochondria to the gap junctions (GJs) of the PM (Forbes and Sperelakis, 1982). The decrease of cell coupling through inhibition of GJs during reperfusion leads to reduced IRI (Rodriguez-Sinovas et al., 2004). In addition, the involvement of GJs has been intensely studied in ischemic preconditioning-induced protection against myocardial reperfusion injury (Miura et al., 2010).

Experimental studies showed that caveolae (PM invaginations which contribute to cell response) are present in cardiomyocyte’s T-tubules located near the mitochondria at the PM (Burton et al., 2017). Consistently, caveolae were also investigated in other studies and found in the cardiomyocytes near the SSM, disclosing a crucial impact on the cell physiology (Fridolfsson et al., 2012). Intriguingly, the mitochondria-associated caveolae number augmented in response to I/R indicating a dynamic relationship linking the mitochondria and the PM (Fridolfsson et al., 2012; Foster et al., 2020). In support of this hypothesis, Fridolfsson and others studied the translocation of caveolin-3 (Cav-3) from the PM to the inner mitochondrial membrane (IMM) in response to cellular stress (Fridolfsson et al., 2012). In the same study, it was shown that Cav-3 overexpression in mouse models provides protection from IRI: cardiac mitochondria exhibit an increase in respiration, improved Ca^2+^ tolerance to higher concentrations and reduced ROS generation. Mice overexpressing Cav-3 showed a reduced infarct size, whereas Cav-3 deletion increased mitochondrial dysfunction. These data suggest a cardioprotective effect of Cav-3 against I/R linked to cell protection and cell adaptation to stress (Fridolfsson et al., 2012). Other studies reported that Cav-1 deletion might also play a protective role in *vivo* I/R mouse models (Tsutsumi et al., 2010). In conclusion, the above described evidences on the involvement of Cav-3 and caveolae in stress response to I/R, might represent an innovative way to counteract tissue damage. Anyway, additional experimental studies are required to unveil the key roles of mitochondria-PM during I/R in mammals.

### Mitochondria-nucleus

As previously described, cardiomyocytes possess different types of mitochondria. Concerning PNM, these mitochondria have different morphology, reduced Ca^2+^ uptake, exhibit increased fusion and fission activity compared to other pools, suggesting different roles among them (Lu et al., 2019). In fact, it has been shown that mitochondrial position inside the cell can control specific Ca^2+^ signaling through a local regulation (Park et al., 2001). Besides Ca^2+^, the perinuclear localization of mitochondria can control nuclear transport across the nuclear envelope, optimizing the distance of energy transfer. Indeed, it ensures the energetic supply necessary for the intense crosstalk nucleus-cytoplasm (Dzeja et al., 2002). The mitochondrial subpopulations are differently associated with different pathological condition. For example it is known that, in the IRI process, OXPHOS is decreased only in SSM type and not IFM one, suggesting the importance of mitochondrial heterogeneity in the pathophysiology of this condition (Lesnefsky et al., 1997).

Mitochondria-nucleus connection is fundamental for the stability of mitochondria: almost all the mitochondrial proteins are encoded by the nucleus and also biogenesis is finely regulated both by mitochondrial DNA (mtDNA) and nuclear DNA (Garesse and Vallejo, 2001). The nucleus controls mitochondrial stability through anterograde signals. Molecular components of this finely regulated system include a number of nuclear genes encoding mitochondrial proteins controlled by different intra- and extra-cellular signals (Ng et al., 2014), in addition, it has been deeply studied the involvement of microRNAs (miRNAs) in the regulation of the nucleus-mitochondria communication. In particular, cardiac tissue is characterized by miR-30 family members (Ikeda et al., 2007) and they have been associated to apoptotic process through regulation of mitochondrial fission machinery by targeting p53-Drp1 axis. miR-30 targets p53 inhibiting Drp1 transcription, thus blocking both mitochondrial fission and apoptosis (Li et al., 2010). Recurring proofs have connected altered miRNA expression to myocardial IRI. Thanks to animal model of I/R and tools of overexpression or inhibition of specific miRNA, it has been defined the opposite role of miRNAs in IRI pathogenesis, or rather, pro-apoptotic or anti-apoptotic acting on different pathways (Yang et al., 2014; Zhu et al., 2018). For instance, *in vitro* experiments in cardiomyocytes have confirmed a role of miR-378 in controlling the level of cell death during ischemia and this miRNA repressed caspase-3 expression reducing both apoptosis and necrosis, thus paving the way to new potential therapies based on miRNA targeting (Fang et al., 2012). A recent study on mice model of I/R has linked miR-138 to hypoxia-inducible factor 1α (HIF-1α). This miRNA showed a cardioprotective role in reducing infarct size and myocardial enzymes, inhibiting expression of cleaved caspase-9, caspase-3 and of HIF-1α (Liu et al., 2019). Another example is given by *in vitro* studies on cardiomyocytes linking increased cardiac damage to the decrease of miR-146a levels after I/R: in normoxic conditions miR-146a localizes to the mitochondria where it modulates CypD protein expression, it prevents mPTP opening and the apoptotic cascade (Su et al., 2021). On the contrary, through knockdown approaches, nuclear miR-762 increase has been associated to apoptosis consequent to I/R: after the treatment, this miRNA translocates to mitochondria, where it interacts with mitochondrial NADH dehydrogenase subunit 2 (ND2) modulating ATP and ROS production and the consequent cardiomyocytes apoptosis (Yan et al., 2019).

It is known that dysfunctions in this inter-organelle communication leads to serious damage to the intricate balance of cellular homeostasis. Indeed, inducers of mitochondrial retrograde signaling such as Ca^2+^-dependent stress, mtDNA damage, metabolical disorders and loss of mitochondrial membrane potential (ψ_m_) permit to impaired mitochondria to communicate with nucleus as a compensative mechanism (Guha and Avadhani, 2013). Recently, new contact sites have been identified: NAM (Nucleus-associated mitochondria) are points of contact of mitochondria with nucleus to favor communication. This retrograde type of communication is mediated by cholesterol, ROS and Ca^2+^ and the tethering between the two organelles is permitted by a complex of different protein such as translocator protein (TSPO), protein kinase A (PKA), A-kinase anchoring protein acyl-CoA binding domain containing 3 (ACBD3), but others might be involved (Desai et al., 2020).

As already explained, one of the main mitochondrial functions is the control of apoptosis through the release of pro-apoptotic factors such cytochrome c and activation of caspase-3 and -9, a mechanism that has been shown to be activated also during reoxygenation after hypoxia in *vitro* experiments on adult cardiomyocytes (Kang et al., 2000). Another mitochondrial effector of apoptotic cell death, independent from the caspases, is Apoptosis Inducing Factor (AIF): it is released from the mitochondria and translocates to the nucleus where it induces chromatin condensation (Susin et al., 1999). The apoptotic role of AIF has been confirmed also in the IRI process. In *vivo* mice hearts subjected to occlusion of the coronary artery, AIF release and nuclear translocation have been considered as a readout of tissue damage. Of interest is the observation that in mice knockout for Hsp70, I/R-induced cardiac dysfunction is amplified, probably because Hsp70 inhibits AIF translocation by direct interaction (Choudhury et al., 2011).

Another type of mitochondria-nucleus communication is through a nuclear cofactor, peroxisome proliferator activated receptor gamma coactivator 1-alpha (PGC-1α), which interacts with both transcription and nuclear factors controlling the relationship between the two organelles. In the IRI condition, PGC-1α is activated by increased ROS and Ca^2+^ and in turn stimulates several mitochondrial related genes, linked to antioxidant system or mitophagy process with a general effect of cardioprotection (Li Y. Q. et al., 2022).

In conclusion, even if the evidences concerning this inter-organellar communication are still limited and more studies are needed, several findings highlighted its importance in the pathophysiology of IRI and in the overall homeostasis of cardiomyocytes.

## Mitochondrial remodeling and state of health in I/R condition

### Fission-fusion

Mitochondria are highly dynamic organelles able to change their number, morphology, and distribution (Tilokani et al., 2018). These changes are coordinated by two crucial events: fission and fusion that define the mitochondrial dynamics in response to the changes in the metabolic status of cardiomyocytes as well as environmental variations. Both processes are pivotal for maintaining mitochondrial integrity and functions, cell cycle modulation and cell quality control (Liu et al., 2020), counterbalancing each other, they are strictly orchestrated by core protein machinery that are mainly large guanosine triphosphates GTPases that exhibit membrane-remodeling characteristics (Lee and Yoon, 2016).

In mammals, mitochondrial fission is coordinated by GTPase Drp1, mitochondrial fission factor (Mff), Fis1, and 49kD and 51kD mitochondrial dynamics proteins (Mid49/51) (Tong et al., 2020). The initiation of mitochondrial fission is mediated by a stringent replication of the mtDNA in the matrix (Lewis et al., 2016). In physiological conditions, mitochondrial fission is relatively less compared with stress conditions, in which Drp1 undergoes post-translational changes including ubiquitination, phosphorylation, acetylation, SUMOylation, and S-nitrosylation (Hu et al., 2020). When activated, Drp1, the master regulator of mitochondrial fission, actively translocates from the cytosol to the OMM surface, where it interacts and binds to its non-GTPases receptors such as Fis1, Mff, and Mid49/51 (Hall et al., 2014).

Mitochondrial fission serves to meet high energy demands: it cleaves a mitochondrion into two daughter mitochondria and it enables clearing the damaged mitochondria from the cardiomyocytes, maintaining the separation of damaged mitochondrial fractions from the whole network. This process is recognized as a prerequisite for selective mitochondrial autophagy termed “mitophagy”, by which damaged mitochondria containing altered proteins, disrupted mtDNA or membranes, are cleared (Suen et al., 2010).

Mitochondrial fusion is regulated by three GTPase dynamin proteins including Mfn1 and Mfn2 and optic atrophy factor 1 (Opa1). The two mitofusins are anchored to the OMM and form homotypic or heterotypic oligodimers to cooperate or work individually to activate OMM fusion with adjacent mitochondria. In contrast, Opa1 is an IMM protein that induces IMM intermingling through the cristae structure regulation (Cohen and Tareste, 2018). Several efforts have been made to define the exact structure and conformational changes of Mfns occurring after phosphorylation, for example through comparative analysis (Mattie et al., 2018) and generation of small-molecule agonists it has been described the specific effect of PINK1 in modulation Mfn2 activity (Rocha et al., 2018).

This dynamic process of fusion consists of two neighboring mitochondria’s OMM and IMM that fuse and share material: this is central for mitochondrial health and physiological functions, including maintaining the balance of matrix metabolites, membrane components and an intact mtDNA (Hoppins et al., 2007).

Mitochondrial remodeling during myocardial I/R involves structural and metabolic changes, both of which are known to play an essential role in each stage of cardiac I/R pathogenesis. During normal physiological conditions, balanced mitochondrial fission with fusion provides an initial step in the culling of defective organelles from the mitochondrial network and facilitates the mitochondrial trafficking to specific subdomains in the membrane inside the cell termed microdomains (Hom and Sheu, 2009; Chang and Reynolds, 2006).

Mitochondrial fission has been shown to be upregulated both during ischemia and reperfusion, when it ultimately decreases ATP production efficiency and disrupts the ψ_m_, thus leading to cardiomyocyte apoptosis (Maneechote et al., 2017). Conversely to mitochondrial fission, when the mitochondrial fusion is downregulated, it promotes mitochondrial fragmentation and rises cardiac cell death in response to I/R (Hall et al., 2016). For example, Bradly and others firstly identified extensive mitochondrial fragmentation during myocardial ischemia in HL-1 cells (Brady et al., 2006). Mitochondrial segregation splits the elongated mitochondrial network into short rods, activating mitochondrial depolarization, ATP depletion, and upregulating ROS generation and apoptosis (Hall et al., 2016). These findings showed that the repression of fission machinery prevents the mitochondrial fragmentation and swelling due to Ca^2+^ overload and were confirmed in various I/R models, both *in vitro*, *ex vivo* and *in vivo*. The increase in mitochondrial fusion reduces the susceptibility to mPTP opening and to cardiomyocytes apoptosis, leading to amelioration of the diastolic, contractile function and reduction in infarct size (Ong et al., 2010; Sharp et al., 2014).

Promoting mitochondrial fusion and repressing excessive mitochondrial fission have been proposed as promising protective mechanisms against myocardial damage after I/R, even if there are some conflicting results in the literature. Indeed, the overexpression of fusion proteins Mfn1 or Mfn2 protects HL-1 cells from IRI through the promotion of mitochondrial fusion (Hall et al., 2016). Also, mitofusins overexpression with the repression of Drp1-mediated fission rescues the cardiomyocytes against the mPTP opening, which is a critical determinant of the cell death process during IRI (Hausenloy and Yellon, 2003; Ong et al., 2010). However, mitofusins deletion increases mitochondrial segregation, thus stimulating the mitochondrial apoptosis in response to I/R (Griparic et al., 2004). Another study, instead, showed that the cardiomyocyte-specific dual deletion of mitofusins reduces cell death after acute myocardial I/R by blocking mPTP activity, thus preventing the mitochondria from elevated Ca^2+^ concentration (Hall et al., 2016). More studies are needed in order to understand the contribution of fission and fusion processes to IRI.

Of note, enhanced expression of mitochondrial calcium uniport (MCU) significantly upregulates Ca^2+^ levels, in return inducing the opening of mPTP and leading to the elevation of calpain expression, which has been shown to inhibit Opa1 expression and to stimulate calcineurin, causing Drp1 phosphorylation and excessive fission in mice models of hypoxia/reoxygenation and myocardial I/R (Patergnani et al., 2020; Guan et al., 2019).

During hypoxia, HIF-1α is a central regulator mediating the cellular response and plays a key role in multiple CVDs, such as IHD (Bouhamida et al., 2022). Interestingly, the expression of the mitochondrial-targeted HIF-1α significantly decreases Drp1 mitochondrial association and Opa1 levels, as well as attenuates apoptosis mediated by hypoxia (Li H. S. et al., 2019). Further experimental studies may provide a better understanding of the molecular mechanisms behind the correlation between the mitochondrial fission-fusion and hypoxia in response to I/R.

The mitochondrial fusion protein Opa1 is intimately involved in the remodeling of cristae and in control of the release of cytochrome c, while its reduction, through the elevation of ROS, aggravates cardiomyocyte apoptosis during I/R (Shen et al., 2007). In support of this finding, Opa1 deficiency has been identified to correlate with I/R-mediated mitochondrial segregation (Ong et al., 2010; Chen et al., 2008) and additionally, Chen and others reported decreased Opa1 levels in human hearts samples with ischemic cardiomyopathy (Chen et al., 2008).

Further insights were obtained using melatonin, a potent mitochondrial-targeted antioxidant (Tan et al., 2016) that induces Opa1 expression at transcriptional level *via* the AMP-activated protein kinase (AMPK) signaling pathway and it increases the mitophagic process, leading to a cardioprotective effect against the IRI (Zhang et al., 2019c).

Pioneering studies have documented the important role of Mff in mediating fatal mitochondrial fission in response to I/R. For instance, Zhou et al. have reported that enhanced levels of nuclear receptor subfamily four group A member 1 (NR4A1) increases, in return, serine/threonine kinase casein kinase two α (CK2α), which promotes Mff mediated-excessive fatal mitochondrial fission, leading to an impaired mitochondrial function and subsequent activation of apoptosis during IRI (Zhou et al., 2018a). In the light of these findings, the higher levels of Mff induced by JNK cause an excessive mitochondrial fission and the activation of mitophagy, thereby triggering cardiomyocyte death (Jin et al., 2018). The phosphorylated Mff elevates the translocation of the cytoplasmic Drp1 into the mitochondria, and the interaction between Mff and Drp1 stimulates mitochondrial segregation, resulting in excessive ROS production, the dissociation of hexokinase 2 (HK2), mPTP opening, and subsequent stimulation of the apoptotic process during cardiac IRI (Zhou et al., 2017).

In addition to the findings mentioned above, Zhang et al. identified that the repression of Socs6, a cyclic AMP-dependent kinase phosphorylation site that inhibits the Drp1 translocation to the mitochondria through the QK/miR-19b/Socs6 signaling pathway attenuates hypoxia/reperfusion-mediated mitochondrial fission and cardiomyocyte impairment *in vitro* and in *vivo* mouse models (Zhang et al., 2022).

Taken together, these findings suggest a major role of mitochondrial fission and fusion in response to myocardial stress such as what occurs during I/R. Thus, the stimulation of mitochondrial fusion factors may have a promising impact on the prevention of myocardial damage during this condition. In spite of the availability of a number of advanced studies focused on the understanding the mitochondrial dynamics, there are only relatively few data correlating I/R in animal models in the regulation of this process. Thus, further insights concerning this issue may provide new therapeutic interventions that are beneficial in cardioprotection.

### Mitochondrial turnover: A balance between biogenesis and mitophagy in IRI

As already mentioned, cell homeostasis requires a fine balance among the three mitochondrial quality control processes: mitochondrial dynamics, already discussed in the previous chapter, biogenesis and mitophagy. Mitophagy is a specific, evolutionary conserved, subtype of macroautophagy, which promotes physiological degradation of senescent or irreversibly damaged mitochondria (Morciano et al., 2020). Therefore, impairment in the mitophagic process, leads to tissue damage, due to accumulation of defective organelles (Morciano et al., 2021a). Canonical mitophagy is controlled by PINK1/Parkin pathway: in summary, under resting condition PTEN-induced putative kinase1 (PINK1) is transported into the IMM where it is degraded by mitochondrial proteases matrix processing peptidase (MPP) and presenilis-associated rhomboid-like protein (PARL) (Jin et al., 2010). Upon loss of ψ_m_, PINK1 accumulates at the OMM to directly recruit Parkin RBR E3 ubiquitin-protein ligase (Parkin) from the cytosol (Koyano et al., 2014), or indirectly by phosphorylation of Mfn2 and promoting Mfn2 Parkin-mediated ubiquitination (Chen and Dorn, 2013). Recent discoveries by Li et al. demonstrate that PINK1-mediated phosphorylation is essential and sufficient, to switch Mfn2 from fusion protein, when not phosphorylated, to mitophagy promoter after phosphorylation on T111, S378 or S442 and that Parkin is not necessary for negative regulation of Mfn2-mediated mitochondrial fusion, but involved in Mfn2 control of mitophagy (Li J. et al., 2022).

Parkin phosphorylation at Ser65 increases its E3 ligase activity, thus promoting poly-ubiquitination of mitochondrial proteins and phagosome recruitment for subsequent protein degradation by the lysosome (Koyano and Matsuda, 2015; Sciarretta et al., 2018). Of note, mutations in the PINK1/Parkin pathway were firstly associated with autosomal recessive Parkinson’s disease (Kitada et al., 1998), but, of interest, extensive studies in cardiovascular diseases highlighted that impairment in PINK1/Parkin pathway promotes alterations in the mitophagic flux leading to cardiac dysfunction. Indeed, mice lacking PINK1 develop cardiac hypertrophy (Billia et al., 2011) and are more susceptible to IRI (Siddall et al., 2013), as well as are associated with cardiac mitochondrial dysfunction and increased ROS production (Billia et al., 2011; Siddall et al., 2013). Moreover, another study on Parkin-deficient mice reported a reduction of mitophagic markers in the border zone of the infarct associated with accumulation of dysfunctional mitochondria (Kubli et al., 2013).

It should be noted that there is a crosstalk among all the mitochondrial quality control mechanisms. For instance, mitochondrial fission is commonly considered a pre-step of mitophagy activation. Downregulation of Drp1 induces, not only mitochondrial fusion, but also an increase of damaged mitochondria due to inhibition of mitophagy, thus worsening myocardial infarction (Ikeda et al., 2015; Song et al., 2015). On the contrary, it has been shown that Opa-1-induced mitophagy mediated cardioprotection against hypoxia-induced apoptosis (Xin and Lu, 2020), and that AMPK-Opa1 signaling pathway induced mitochondrial fusion/mitophagy upon melatonin treatment thus attenuating cardiomyocyte death and mitochondrial stress in the setting of cardiac IRI (Zhang et al., 2019c). According to that, cardiac myocytes overexpressing Parkin, are more protected against hypoxia-mediated cell death. These data suggest that a mild induction of mitophagy in myocardial infarction has a beneficial role to clear dysfunctional mitochondria, whereas chronic activation of mitophagy may be detrimental leading to chronic heart diseases such as hypertrophy and heart failure (Shires and Gustafsson, 2015; Ramaccini et al., 2020; Morciano et al., 2020).

In addition, mitophagy may also occur through the ubiquitin-independent mechanism, mediated by adaptor proteins located at the OMM which contain an LC3 interacting motif, thereby recruiting autophagosomes to damaged mitochondria. These receptors include: BNIP3 (BCL2/Adenovirus E1B 19 KDa Protein-Interacting Protein 3), BNIP3L, also known as NIX, (BCL2 interacting protein three like) (Zhang and Ney, 2009; Zhang and Ney, 2011; Lee et al., 2011) and FUNDC1 (Liu et al., 2012). Several studies showed the role of these proteins in acute I/R by modulating mitophagy, but the role of BNIP3 and NIX in the heart is still unclear, since these two proteins exhibit both the capacity to induce cell death and to promote autophagy (Zhang and Ney, 2009).

It has been reported that upon hypoxia, mitophagy is upregulated through activation of the HIF-1α/BNIP3 axis, promoting cardioprotection by removing damaged mitochondria and promoting myocardial remodeling after IRI (Zhang et al., 2008; Bouhamida et al., 2022; Hamacher-Brady et al., 2007; Zhang et al., 2019b). However, other studies suggested that after cardiac stress, overexpression of BNIP3 induces cardiomyocytes death and a decreased myocardial function (Regula et al., 2002; Diwan et al., 2007). With regard to the autophagic cell death role, it is unclear whether death is due to excessive autophagy or to an independent death-inducing function of BNIP3 or NIX. FUNDC1 interacts with LC3 upon hypoxia in ischemic heart, promoting mitophagy, which is associated with inhibition of apoptotic cell death; however, after reperfusion, FUNDC1-mediated mitophagy is inactivated, resulting in a boost of cell death (Liu et al., 2012; Zhou et al., 2018b).

Dysfunctional mitochondria are cleared by mitophagy, whereas mitochondrial population and mass are maintained by activation of mitochondrial biogenesis (Palikaras et al., 2015). Mitochondria cannot be synthesized *ex novo*, and in resting conditions, mitochondrial turnover in the adult heart occurs every 2 weeks (Dorn et al., 2015). Moreover, mitochondrial biogenesis is activated and increased in response to high energy demands, such as right after exercise or cold, or under stress conditions, such as hypoxia and oxidative stress (Ham and Raju, 2017). Mitochondria are unique and semiautonomous organelle, which contain their own mtDNA. However, mtDNA encodes only 23 essential subunits of the electron transport chain (ETC), as well as all rRNAs and tRNAs. Therefore, mitochondrial biogenesis requires a fine-tuned coordination of four processes: transcriptional activation of nuclear-encoded mitochondrial genes, mitochondrial protein translocation, replication of mtDNA and synthesis of mitochondrial phospholipids (Sun et al., 1990; Dorn et al., 2015).

To date, the mechanisms controlling the mitochondrial biogenesis process are not fully understood. Several transcriptional factors are involved in this process and PGC-1α is considered the master regulator of mitochondrial biogenesis with a key role also in oxidative metabolism and antioxidant defenses in mammalian cells (Fernandez-Marcos and Auwerx, 2011). Briefly, during mitochondrial biogenesis, PGC-1α translocates into the nucleus where it co-activates nuclear respiratory factors 1 and 2 (NRF1 and NRF2), which are regulators of the expression of subunit of ETC’s complexes, or mitochondrial transcription factor A (TFAM) and mitochondrial transcription factor B2 (TFB2M), required for transcription and translation of mitochondrial genes (Gleyzer et al., 2005; Scarpulla, 2008; Ding et al., 2021). Other transcriptional factors that are activated by PGC-1α are peroxisome proliferator-activated receptor (PPARβ) and estrogen receptor-related receptor-alpha (ERRs), which are mainly involved in energy metabolism regulation (Giguere, 2008).

Several studies have reported downregulation of PGC-1α expression in the failing heart, which is commonly associated with low ATP production and reduced energy metabolism (Pisano et al., 2016; Riehle et al., 2011). Mitochondrial biogenesis is also reduced in cardiac IRI: recent studies reported that PGC-1α is induced by hypoxia as an adaptive mechanism that increases cardiac regeneration, thus facilitating the recovery of infarcted heart (Sun et al., 2007; Honda et al., 2008; Yue Tl et al., 2001). On the same line, Andres et al. propose the detection of PGC-1α in blood samples as a prognostic factor during myocardial infarction (Fabregat-Andres et al., 2011; Fabregat-Andres et al., 2016). They reported that peripheral levels of PGC-1α correlate with expression in cardiac muscle, which are inversely related to cardiac recovery after IRI (Fabregat-Andres et al., 2011; Fabregat-Andres et al., 2016; Fabregat-Andres et al., 2015). Therefore, PGC-1α might represent an interesting target due to its cardioprotective role in myocardial infarction.

An interesting study reports that melatonin is capable to induce AMPK activation (Qi and Wang, 2020), which simultaneously inhibits mammalian target of rapamycin (mTOR) and induces PGC-1α activation, stimulating mitophagy and mitochondrial biogenesis respectively (Xu et al., 2012; Kupr and Handschin, 2015), ultimately protecting the injured heart.

It is interesting to note that the Hippo pathway, well studied in other pathological conditions but just lately come to light into the cardiovascular field, has been found to be less active in cardiac regeneration, while upstream kinases of the pathway are elevated in myocardial infarction (Ramaccini et al., 2022). According to that, a recent work by Chen et al., showed that the deletion of large tumor suppressor kinase 2 (LATS2), an upstream kinases of YAP1, increased cardiomyocyte viability and mitochondrial biogenesis in a PGC-1α-dependent manner, by increasing expression levels of PGC-1α, in association to the rise of mRNA levels of TFAM and NRF1 in cardiomyocytes (Chen et al., 2021). In this scenario, LATS2 suppression ameliorates mitochondrial functions and attenuates myocardial infarction.

In conclusion, both mitophagic process and biogenesis are fundamental for mitochondrial health and general cellular homeostasis, and stimulation of these mechanisms might become a cardioprotective strategy to challenge IRI.

### mPTP: A leading role in IRI

Of great interest to all the scientific community, the molecular composition of the mPTP has been explored widely over the past 40 years, and different hypotheses have been proposed and retracted during the years. The purpose of this review is not to gather the most recent findings on mPTP structure, but to focus on the contribution of this multiprotein complex in IRI.

In summary, mitochondrial permeability transition induces the opening of two types of pores characterized by different conductance: a low conductance pore that is associated to adenine nucleotide translocator (ANT) (Neginskaya et al., 2019; Karch et al., 2019; Carrer et al., 2021); a high conductance pore linked to F_1_F_O_-ATP synthase and its dimer formation or disassembly (Bonora et al., 2017), or the conformational changes of its c-ring (Bonora et al., 2013). These two pores work in balance and this is confirmed by the direct interaction of ANT and F_1_F_O_-ATP synthase: the low conductance state is a transitory opening involved in Ca^2+^ homeostasis, whereas the high conductance state is long-lasting and leads to cell death (Wacquier et al., 2020). An important regulator of mPTP is CypD (a peptidyl-prolyl cis-trans isomerase) (Halestrap et al., 1997), which interacts with oligomycin sensitivity conferral protein (OSCP) of F_1_F_O_-ATP synthase (Giorgio et al., 2017): this is confirmed by the most known inhibitor of mPTP, cyclosporine A (CsA), which targets cyclophilin D (CypD). In addition to CypD, several regulators of mPTP have been recognized to be positive or negative actors in this process, either through interaction or post-translational modifications (Bonora et al., 2022).

Pathophysiology of ischemia/reperfusion at cellular level has been robustly elucidated by mPTP contribution in conditioning cell damage (Hausenloy et al., 2020; Morciano et al., 2015), since a bunch of paper and reviews have focused on this aspect. The objective of this subchapter is to give an overall point of view of the process with a perspective on recent studies.

The lack of oxygen after the ischemic event leads to cardiomyocyte dysfunction linked to mitochondrial ability: the ETC reduces its activity and the production of ATP, essential for all metabolic processes (Hausenloy and Yellon, 2013). To counteract energy shortage, cells switch to anaerobic glycolysis causing the rise of lactate and hydrogen ions and consequent cellular acidosis. In healthy conditions, the reestablishment of pH is carried out by PM Na^+^/H^+^ exchanger and the consequent accumulation of cytosolic Na^+^ is compensated by Na^+^/K^+^ ATPase, which uses cellular ATP reserves: lack of energy, in the form of ATP, blocks the activity of, not only Na^+^/K^+^ ATPase, but also plasma membrane calcium ATPase (PMCA) and SERCA, and the final outcome is an overload of cytosolic Ca^2+^ (Piper et al., 2003; Bonora et al., 2019). Of note, during ischemia mPTP is closed, inhibited by an increase in the ADP/ATP ratio and by low pH. It is known that the opening of mPTP is optimal at pH 7.4 (Haworth and Hunter, 1979), whereas at pH 6.5 the channel is blocked due to the protonation of the highly conserved His112 of OSCP subunit of F_1_F_O_-ATP synthase (Antoniel et al., 2018). Only after few minutes of reperfusion, oxygen recover allows restoration of mitochondrial respiration, ATP production and pH neutralization but also ROS production (Granger and Kvietys, 2015), which stimulates mPTP opening and Ca^2+^ accumulation in mitochondria (Griffiths and Halestrap, 1995). As already mentioned, the opening of this mitochondrial pore leads to ψ_m_ dissipation, ATP production arrest, mitochondrial swelling and finally activation of regulated cell death process (Halestrap et al., 2004; Ong and Gustafsson, 2012; Bonora et al., 2020).

A very recent publication has linked Mitofilin, an IMM protein with function of control of mitochondrial structure and remodeling, to IRI. In particular, it has been shown that mitofilin binds to CypD through its C-terminal (Baines et al., 2005; Nakagawa et al., 2005; Schinzel et al., 2005). This interaction is reduced during the early moments of reperfusion, specifically, after I/R, Mitofilin-CypD interaction is disrupted and the protein levels decrease with an inversely proportional trend to the degree of myocardial infarct size (Tombo et al., 2020). In addition, the amount of myocardial injury on an *in vivo* I/R model has been correlated to CypD phosphorylation on its S191 residue, which regulates its ability to control mPTP opening and to interact with OSCP (Hurst et al., 2020).

In the past, ANT was suggested as a hypothetical pore-forming component of the mPTP but this evidence has been later rejected. This protein, instead, is an important regulator of mPTP (Karch and Molkentin, 2014). Indeed, mitochondria extracted from hearts of rats overexpressing ANT1, the main isoform expressed in the heart, showed a strong cardioprotection against IRI in terms of mitochondrial Ca^2+^ retention capacity and ψ_m_, indexes of mPTP opening and mitochondrial oxygen consumption (Dorner et al., 2021). In a previous study, the same group reported that this ANT1-overexpressing *in vivo* model was strictly related to augmented cell survival of hypoxic cardiomyocytes and this was probably due to the fact that ANT1 controlled oxidative stress by decreasing ROS generation thus protecting from IRI (Klumpe et al., 2016).

Several studies have connected the c subunit of F_O_-ATP synthase to mPTP functionality and pore formation (Bonora et al., 2013; Azarashvili et al., 2014; Alavian et al., 2014), with particular involvement in the IRI process. Serum levels of c subunit protein correlate with several surrogate markers of myocardial reperfusion in STEMI patients (Campo et al., 2016), and selective compounds targeting the c subunit inhibit mPTP opening and IRI in animal models, ameliorating cardiac function and reducing apoptosis (Morciano et al., 2018; Fantinati et al., 2022). In addition, a mutation in the glycine-rich domain of c subunit, a highly conserved domain, leads to functional and conformational impairments (Huang and Docampo, 2020; Alavian et al., 2014). In particular, in STEMI patients a mutation that aggravates mPTP-mediated IRI when expressed in human cardiomyocytes has been found (Morciano et al., 2021b). Besides mutations, a significative and positive correlation between the mPTP opening measured in fibroblasts from patients and IRI degree, evaluated by the cardiac magnetic resonance imaging, was found (Morciano et al., 2022).

In support of this model based on mPTP, *in vitro* studies from different groups have shown that ATP synthase inhibitory factor subunit 1 (ATPIF1) overexpression promotes stabilization of F_1_F_O_-ATP synthase dimers and consequent inhibition of mPTP opening (Bonora et al., 2017; Garcia et al., 2006). I/R experiments *in vitro* and *in vivo* showed an upregulation of this protein in cardiomyocytes and hearts. Finally, its overexpression through adenovirus or AAV9 vector protected against cardiac dysfunction induced by I/R *ex vivo* and *in vivo* conferring cardioprotection (Wu et al., 2022).

As already pointed out, the ischemic event leads to activation of HIF-1α, which is able to activate mechanisms of safeguard of the heart from IRI (Ong and Hausenloy, 2012). HIF-1α stabilization using pharmacological and genetic approaches resulted in cardioprotection as a consequence of inhibition of mPTP opening and of the increase of the protein levels of the glycolytic enzyme HK2, a known negative regulator of mPTP, which interacts with VDAC (Ong et al., 2014; Bonora et al., 2022).

Of note, mPTP involvement in IRI is the most studied process among those previously described. Thus, several pharmacological approaches of inhibition of its opening are already in use both in preclinical and clinical studies. Studies, however, concerning the composition of this multiprotein complex are still necessary.

## Recent clinical strategies based on targeting mitochondria

As previously discussed, adult cardiomyocytes lack of effective renewal ability. Thus, after IRI, cardiac tissue endures a physiopathological remodeling that cannot be restored by existing clinical therapy. Given the important involvement of mitochondrial activity in worsening of cardiac damage after I/R, several mitochondrial-targeted strategies has been developed in order to reduce cardiac IRI (summarized in Table 1 and in Figure 5).

**TABLE 1:** Summary of recent therapies based on targeting mitochondria in I/R.

Mitochondrial target	Pharmacological agent/therapy	Study design	Mechanism of action/effect	References
mPTP opening: CypD	CsA	*In vivo* in male Sprague-Dawley rats	Reduces infarct size and IRI	Xie and Yu (2007)
		Porcine model of I/R	No reduction in infarct size	Karlsson et al. (2010), Karlsson et al. (2012)
		Randomized controlled trial	Positive effects when administered at reperfusion during acute myocardial infarction	Piot et al. (2008)
		Clinical trials: NCT01502774	Fails to cardioprotect, no improvement in clinical outcomes of STEMI patients	Cung et al. (2015)
		NCT01650662		Ottani et al. (2016)
		Randomized controlled trial		Ghaffari et al. (2013)
mPTP opening: CypD	SS31	*In vitro* on isolated mitochondria and *in vivo* in male Dorsett hybrid sheep and male New Zealand White rabbits, *ex vivo* in guinea pig I/R models	Targets the IMM, binds to cardiolipin and reduces ROS	Zhao et al. (2004), Kloner et al. (2012)
		Clinical trial with STEMI patients (EMBRACE STEMI)	No reduction in myocardial infarct size	Gibson et al. (2016)
mPTP opening: CypD	CsA@PLGA-PEG-SS31	*In vitro* in H9c2	Decreases cell death by inhibiting mPTP activity and apoptotic cascade activation	Zhang et al. (2019a)
		*In vivo* in male Sprague–Dawley rats I/R models	Reduces myocardial infarction	
mPTP opening: CypD	Combination of CsA and Pitavastatin nanoparticles	*In vivo* in male C57BL/6J mice I/R models	Targets mPTP and inflammation, reduces the infarct size	Ikeda et al. (2021)
mPTP opening: CypD	Sanglifehrin A (SfA)	*In vivo* in female Sprague-Dawley rats	Cardioprotection only in early reperfusion time	Parodi-Rullan et al. (2018)
mPTP opening: CypD	C31	*In vitro* in H9c2, adult mouse cardiomyocytes and isolated mouse cardiac mitochondria	Inhibited CypD peptidylprolyl *cis-trans* isomerase (PPIase) activity and mitochondrial swelling	Panel et al. (2021)
		*Ex vivo* in male C57BL/6J mice I/R models	No effect after systemic administration	
mPTP opening	AP39	*In vitro* in H9c2 and isolated cardiac mitochondria, *in vivo* in male Wistar rats and C57BL/6J mice I/R models	Inhibits mPTP and reduces infarct size	Karwi et al. (2017), Chatzianastasiou et al. (2016)
mPTP opening: Oxidative stress	Lycopene (LP)	*In vitro* in H9c2, *ex vivo* in male Wistar rats I/R models	Blocking mPTP activity and the mitochondrial apoptotic cascade through the modulation of Bax and Bcl-2	Li et al. (2019b)
mPTP opening: Oxidative stress	SS31-peptide-resveratrol	*In vitro* in H9c2, *in vivo* in I/R rats	Reduces of mitochondrial ROS, inhibits mPTP opening, and decreases apoptosis, reduces infarct size	Cheng et al. (2019)
mPTP opening: c subunit of ATP synthase	1,3,8-triazaspiro [4.5]decane (PP11)	*In vitro* in HeLa, *ex vivo* in Wistar rats I/R models	Decreases the apoptotic cell death	Morciano et al. (2018)
			Improves cardiac function after I/R	
Succinate oxidation	Chloramphenicol succinate (CAPS)	*In vivo* in porcine heart	Reduces IRI and increases autophagy	Sala-Mercado et al. (2010)
Oxidative stress	MitoSNO	*In vivo* in C57BL/6J mice I/R model	Reduction in ROS production, oxidative damage and IRI.	Chouchani et al. (2013), Prime et al. (2009)
		*In vitro* in H9c2		
Oxidative stress	Amobarbital	*Ex vivo* in male Fisher rats, mice and rabbits I/R model	Protects mitochondria against ROS overproduction and ischemic damage, blocks of electron transport, decreases mPTP opening, protects against apoptosis during I/R	Chen et al. (2006), Chen and Lesnefsky (2011), Xu et al. (2013)
Oxidative stress	S1QELs	*In vitro* in H9c2 and *ex vivo* in male C57BL/6J mice I/R models	Destroy superoxide-H_2_O_2_ production at complex I and their correlated damage	Brand et al. (2016)
Oxidative stress	KAI-9803 (δPKC inhibitor peptide)	*In vivo* porcine model of acute myocardial infarction	Decreases apoptosis and necrosis	Inagaki et al. (2003)
		Clinical trial with Acute Myocardial Infarction patients (DELTA MI)	Shows an acceptable safety and tolerability profile but no reduction of biomarkers of IRI	Bates et al. (2008)
		Clinical trial NCT00785954		Lincoff et al. (2014)
Oxidative stress	MitoQ	*In vivo* in male Wistar rat I/R models	Accumulates in the mitochondrial matrix, decreases oxidative injury, ameliorates cardiac function reducing tissue damage	Adlam et al. (2005)
		Mouse model of heterotopic heart transplantation	Reduces graft damage *via* oxidative stress inhibition	Dare et al. (2015)
Oxidative stress	N-[(R)-1,2-dithiolane-3-pentanoyl]-L-glutamyl-l-alanine (CMX-2043)	*In vivo* in male Sprague-Dawley rat I/R models	Decreases infarct damage both before and during the ischemic injury and at reperfusion	Baguisi et al. (2016)
Mitochondrial biogenesis: PPARs	Bezafibrate	Randomized controlled trial (BIP)	Reduces major cardiac events and mortality	Madrid-Miller et al. (2010), Goldenberg et al. (2008), Goldenberg et al. (2009)
Mitochondrial biogenesis: PPARs	Thiazolidinediones (TZDs), Pioglitazone	Randomized controlled trial	Decreases cardiovascular deaths, non-fatal myocardial infarction	Liu and Wang (2017)
Mitochondrial biogenesis: PPARs	Rosiglitazone	*In vivo* in swine models and male rat model	Fails to prevent the ΔΨ_m_ collapse, nor reduces the mitochondrial ROS. No positive effect on cardiac function	Palee et al. (2011)
		Meta-analysis on clinical trials	Associated to potential serious adverse cardiovascular effects	Nissen and Wolski (2007)
Mitochondrial biogenesis: AMPK agonist	5-aminoimidazole-4-carboxamide ribonucleoside (AICAR)	*In vitro* in neonatal rat cardiac myocytes	Protects against ischemic insult	Sunaga et al. (2019)
Mitochondrial biogenesis: AMPK agonist	Metformin	*In vitro* in human-induced pluripotent stem cell-derived cardiomyocytes (hiPSC-CMs) and in mitochondria isolated from human cardiac tissue	A biphasic impact: at low concentration, increases OCR and mitochondrial biogenesis; at high concentration, increases glycolysis, attenuates superoxide production and inhibits Ca^2+^-induced mPTP opening	Emelyanova et al. (2021)
		Murine cardiac acute and chronic transplantation models	Reduces cardiac rejection and limit acute IRI	Chin et al. (2011)
		*In vivo* in male Wistar rat I/R models	Improves cardiac function following the reduction of mitochondrial fission, ROS production, apoptosis, and mitochondrial swelling	Palee et al. (2020)
		Randomized controlled trials	Contradictory results	Holman et al. (2008), Mellbin et al. (2008), Hartman et al. (2017)
mPTP opening	Melatonin	*In vitro* in neonatal cardiac microvascular endothelial cells, *in vivo* in Sprague–Dawley rat I/R models	Inhibits mPTP opening, inhibits autophagy *via* AMPK/mTOR signaling. Reduces cardiac damage	Chen et al. (2018)
Oxidative stress				
Mitochondrial fusion	M1	*In vivo* in male Wistar rat I/R models, *in vitro* in embryonic fibroblasts (MEFs)	Decreases infarct size and cardiac apoptosis	Maneechote et al. (2019)
Mitophagy	Rapamycin	*In vivo* in male Wistar rat I/R models, *in vitro* in H9c2	Promotes autophagy, reduces cardiomyocyte apoptosis and infarct size after I/R	Gao et al. (2020)
Mitophagy: Inhibition of mTORC1	Everolimus	*In vivo* in male Wistar rat I/R models	Prevents left ventricular remodeling after myocardial infarction	Buss et al. (2009)
Mitochondrial fission: Drp1	Mdivi-1	*In vitro* in murine neonatal cardiomyocytes, *ex vivo* in male Sprague-Dawley rat I/R models	Preserves OCR and cardiomyocytes function, improves diastolic function	Sharp et al. (2014)
		*In vitro* in HL-1 and adult murine cardiomyocytes, *in vivo* C57BL/6 male mice I/R models	Decreases mPTP sensitivity and reduces cell death after I/R, reduces infarct size	Ong et al. (2010)
		*In vivo* C57BL/6 male diabetic mice I/R models	Reduces the troponin I levels, lactate dehydrogenase activity, blocks mPTP opening, attenuates cardiac injury	Ding et al. (2017)
		*In vitro* in HL-1	Pre-ischemic treatment shows cardioprotection, treatment during reoxygenation upregulates to necroptosis	Dong et al. (2016)
		*In vivo* swine model of myocardial infarction	Treatment at the onset of reperfusion do not reduce infarct size nor ameliorate cardiac function	Ong et al. (2019)
Mitochondrial fission: Drp1	Drpitor1 and Drpitor1a	*Ex vivo* in male Sprague-Dawley rat I/R models	Decreases ROS production and preserves diastolic function	Wu et al. (2020)
Mitochondrial fission: Drp1	P110	*In vitro* rat primary cardiomyocytes, *ex vivo* rat I/R model, and *in vivo* in male Wistar rat I/R models	Improves mitochondrial O_2_ consumption and cardiac function, reduces autophagy and apoptosis	Disatnik et al. (2013)
Mitochondrial fission: Drp1	Hydralazine	*In vitro* in mouse embryonic fibroblasts and ventricular cardiomyocites. *In vivo* and *ex vivo* in C57/BL6 mouse I/R model	Reduces cardiomyocyte death and infarct size	Kalkhoran et al. (2022)
Mitochondrial fission: Drp1	miR-499	*In vitro* in neonatal rat cardiomyocytes and *in vivo* in rat and mice I/R model	Inhibits apoptotic pathway and ameliorates cardiac function	Wang et al. (2011)
Autophagy	EPO	*In vitro* in rat ventricular cardiomyocytes *In vivo* in male Sprague-Dawley rat I/R models	Reduces sensitivity of mPTP to ROS, reduces infarct size attenuating ventricular damage	Ahmet et al. (2011)
		*In vitro* in H9c2	Increases cell viability, decreases autophagy through activation of PI3K/Akt pathway	Lin et al. (2020)
Mitochondrial functionality	Mitochondria transplantation	*In vitro* in neonatal rat cardiomyocytes *In vivo in* New Zealand White rabbit I/R models	Reduces markers of myocardial injury and infarct size, reduces inflammatory markers and necrosis	Masuzawa et al. (2013)
		*Ex vivo in* New Zealand White rabbit I/R models	Perfusion through the coronary vasculature leads to reduction of infarct size and enhanced post-ischemic myocardial function	Cowan et al. (2016)
		Pediatric patients who required central extracorporeal membrane oxygenation support for I/R associated myocardial dysfunction after cardiac surgical procedure	No short-term adverse reactions, improves ventricular function	Emani et al. (2017)
		Pediatric patients who required central extracorporeal membrane oxygenation support for cardiogenic shock due to IRI after cardiac surgery	No arrhytmias, no inflammatory or immune response, enhances ventricular strain	Guariento et al. (2021)
		*In vitro* in H9c2 *In vivo* in male Sprague-Dawley rat I/R models	Reduces inflammation and oxidative stress and protects mitochondrial integrity, ameliorates cardiac function	Lee et al. (2018)

* Metformin Confers Cardiac and Renal Protection in Sudden Cardiac Arrest *via* AMPK Activation. Cody A. Rutledge, Claudia Lagranha, Takuto Chiba, Kevin Redding, Donna B. Stolz, Sunder Sims-Lucas, Cameron Dezfulian, Jonathan Elmer, Brett A. Kaufman bioRxiv 2021.08.24.457506; doi: https://doi.org/10.1101/2021.08.24.457506).

### Mitochondrial target: mPTP opening

As already discussed in subchapter 3d, mPTP opening during the first minutes of reperfusion contributes to IRI, triggering processes of apoptosis and necrosis. Thus, several studies have focused on inhibiting mPTP directly acting on its regulators. Among them, targeting CypD has been extensively addressed in developing cardioprotective strategies: the use of CsA *in vivo* has been shown to reduce infarct size and IRI in small animal models (Xie and Yu, 2007) but failed in porcine model of I/R (Karlsson et al., 2010; Karlsson et al., 2012). In addition, the initial clinical studies showed positive effect of CsA administered at reperfusion in patients during acute myocardial infarction (Piot et al., 2008), but subsequent clinical trial have given contradictory results (Cung et al., 2015; Ottani et al., 2016; Ghaffari et al., 2013). Indeed, an important side effect of CsA is its systemic immunosuppressant activity. It is important to note that an effective drug treatment should deliver the compound directly to the mitochondria of the myocardium. Very recently, SS31 (Szeto, 2014), a small cell-permeable peptide, able to selectively target mitochondria at IMM, has been tested. This compound has shown to be effective *in vitro* on isolated mitochondria and in animal models of I/R, reducing ROS levels (Szeto, 2008; Zhao et al., 2004; Kloner et al., 2012). The direct target of SS31 is not as yet recognized, but its protective action might be associated to its binding to cardiolipin (Birk et al., 2013), a phospholipid existent on IMM fundamental for cristae formation and bioenergetic efficiency (Frey and Mannella, 2000; Kiebish et al., 2012; Allen et al., 2020). Also, this peptide has been tested in a human clinical trial with STEMI patients, the EMBRACE STEMI multicenter phase IIa trial, with unsatisfying results: the peptide is safe and well tolerated but it failed to reduce myocardial infarct size (Gibson et al., 2016).

Thanks to nanoparticle technology and to this specific peptide, researchers designed a different approach to intensify CsA performance: CsA@PLGA-PEG-SS31 nanoparticle delivers CsA directly into the mitochondria of ischemic cardiomyocytes (Zhang C. X. et al., 2019). CsA@PLGA-PEG-SS31 delivering CsA into mitochondria in *vitro* experiments of I/R, reduced cell death, inhibited mPTP opening and apoptotic cascade activation. These effects were confirmed *in vivo,* resulting in the reduction of myocardial infarct area. Other approaches with nanoparticles carrying CsA were made in order to find a drug delivery system specific to cardiac injured tissue with encouraging results (Ikeda et al., 2016). Lately, in an attempt to develop polymeric nanoparticles containing CsA, it has been combined with pitavastatin, in order to target simultaneously mPTP opening and monocyte-mediated inflammation: this approach resulted in cardioprotection after a long duration of ischemia in mice, avoiding the increase of oxidative stress (Ikeda et al., 2021). Another CypD inhibitor studied is Sanglifehrin A. This compound showed positive effects on female rat hearts only early after acute myocardial infarction and not at 28-day post-infarction, indicating a cardioprotective action of CypD inhibition only in early reperfusion time when mPTP opening is significant (Parodi-Rullan et al., 2018).

Very recently, a new non-peptidic small-molecule CypD inhibitor, C31, has been shown to be effective on mPTP opening in isolated cardiac mitochondria, but, in an *ex vivo* model of I/R, its effect was found only at relatively high concentrations. In addition this molecule failed to reach cardiac mitochondria in living mice (Panel et al., 2021).

AP39 is a mitochondrial-targeted, triphenylphosphonium (TPP)-bound donor of H_2_S, with cardioprotective effects, shown both *in vitro* and *in vivo* in mice models of I/R. AP39 reduced infarct size, dose-dependently, by inhibiting mPTP, independently from CypD even if the exact target of mPTP remains to be elucidated (Karwi et al., 2017; Chatzianastasiou et al., 2016).

Lycopene (LP) is a well-known potent antioxidant which exerts also protective roles against IRI both *in vitro* and *ex vivo*, by acting on inhibition of mPTP and the consequent activation of mitochondrial apoptotic cascade (Li X. et al., 2019). Similar data were obtained with another antioxidant, resveratrol: to avoid its rapid metabolism and taking advantage of nanoparticle technology, has been developed an ischemic myocardium targeted peptide, SS31-peptide-resveratrol. These nanoparticles delivered to cardiac mitochondria in the ischemic tissue induced a decrease of mitochondrial ROS, inhibition of mPTP opening and reduction of apoptosis *in vitro*, whereas *in vivo* experiments confirmed the accumulation of resveratrol in ischemic myocardium with a concomitant reduction in infarct size (Cheng et al., 2019). This technological approach, e.g. the development of nanoparticles carrying bioactive peptides capable to deliver compound to a specific tissue or even organelle, is of great relevance, due to its capacity to ameliorate the pharmacokinetic properties and metabolism of the drugs delivered (Forini et al., 2020).

All the above-mentioned approaches sometimes failed in clinical studies probably because they do not target the specific proteins involved in mPTP opening but only regulatory proteins. As already enhanced, c subunit of F_O_-ATP synthase plays a pivotal role in mPTP activity and formation, therefore targeting specifically this protein might be a new effective approach in IRI treatment. In fact, recently, a novel class of small molecules targeting c subunit has shown beneficial effects *in vitro* and in an *ex vivo* model of myocardial infarction, with a reduction of apoptotic cell death upon IRI; of note, these molecules localize specifically to mitochondria avoiding side-effects in other cellular compartments and do not affect mitochondrial ATP content or production even if they target a component of ATP synthase (Morciano et al., 2018).

### Mitochondrial target: Oxidative stress

During ischemia succinate levels increase and after reperfusion this accumulated succinate is quickly re-oxidized by succinate dehydrogenase (SDH), inducing extensive ROS production by reverse electron transport at mitochondrial complex I (Chouchani et al., 2014; Chouchani et al., 2016). Hence, potential therapeutic strategies have been focused on avoiding ischemic succinate accumulation and on reducing its oxidation after reperfusion with a consequent impact on global myocardial IRI. For example, chloramphenicol succinate (CAPS) is a prodrug which may be hydrolyzed by esterase or maybe a competitive oxidative substrate for SDH (Ambekar et al., 2004). An *in vivo* study on porcine heart revealed that CAPS administration, as a pretreatment or before reperfusion, reduces IRI in association with increased myocardial expression of molecular markers of autophagy like Beclin-1 and LC3-II (Sala-Mercado et al., 2010). This association, although of interest, up to now does not allow to conclude that there is causality between the two phenomena.

As previously described, succinate accumulation leads to uncontrolled superoxide production by complex I that is the main font of ROS during IRI (Chouchani et al., 2014). Indeed, pharmacological tools designed against damaging reactive species specific for respiratory complex I have led to promising results even if only at pre-clinical stage. *In vivo* systemic administration at low doses before reperfusion of MitoSNO, a mitochondria-selective S-nitrosating agent that acts on complex I S-nitrosation of Cys^39^ on the ND3 subunit, during an ischemic event, showed a reduction in ROS production, oxidative damage and IRI (Chouchani et al., 2013; Prime et al., 2009).

Using Langendorff-perfused rat hearts, the reversible blockade of respiratory chain during ischemia with amobarbital, an inhibitor at the rotenone site of complex I, has been associated to protection of mitochondria against ROS overproduction and consequent ischemic damage (Chen et al., 2006). In addition, the blockade of electron transport protects mitochondria during ischemia also avoiding Bcl-2 depletion, decreasing mPTP opening (Chen and Lesnefsky, 2011), preventing both AIF and cytochrome c release from mitochondria and reducing both caspase-dependent and independent apoptotic pathways during I/R (Xu et al., 2013).

Others small molecule inhibitors of complex I, which are specifical and selective suppressors of ubiquinone (Q)-binding site (site I_Q_) (S1QELs), have shown both *in vitro* and *ex vivo* encouraging results in destroying superoxide-H_2_O_2_ production at complex I and the correlated damage, without affecting ψ_m_ and resting OXPHOS (Brand et al., 2016).

δPKC is a serine/threonine protein kinase involved in the apoptotic reaction of cells to various stimuli. This protein is a cytosolic redox-sensitive molecule that moves to mitochondria triggering apoptotic cascade through the release of cytochrome c and the activation of caspase 3 (Majumder et al., 2000). In addition, it has been shown that δPKC is activated by oxidative stress and in turn augments mitochondrial superoxide anion production during reperfusion (Churchill and Szweda, 2005). Experiments on an *in vivo* porcine model of acute myocardial infarction defined a cardioprotective effect of a δPKC inhibitor peptide (KAI-9803) when administrated at the time of reperfusion: the outcomes are smaller infarct size and increased cardiac function, due to reduction of apoptosis and necrosis (Inagaki et al., 2003). A randomized controlled trial has been initiated, in order to define the safety and tolerability profile of the delivery of this compound *via* intracoronary injection during primary percutaneous coronary intervention for STEMI (Bates et al., 2008). Unfortunately, a subsequent clinical trial did not confirm the inhibitory effect on myocardial injury (Lincoff et al., 2014).

Of note, the most logical approach to limit the damage after I/R is to act on oxidative stress. For example, very promising results derived from MitoQ, a well-studied mitochondria-targeted antioxidant already tested in different phase II clinical studies (NCT00329056 and NCT00433108). Because of its link with a lipophilic cation triphenylphosphonium (TPP), it crosses the phospholipid bilayers and accumulates in mitochondrial matrix, where it carries out its action: it is a bioactive ubiquinone compound which is reduced into the antioxidant ubiquinol, thus reduces oxidative injury (Smith and Murphy, 2010). This compound had also a cardioprotective effect *in vivo* in rat hearts subjected to I/R ameliorating cardiac functional parameters and reducing tissue damage through a defensive action on mitochondria (Adlam et al., 2005). As already mentioned, IRI is a predictable consequence of organ transplantation, thus, the use of these above described classes of compounds also in this condition might improve graft function and survival. An example is given by MitoQ, since its administration in a mouse model of heterotopic heart transplantation reduces graft damage as the consequence of the inhibition of oxidative stress (Dare et al., 2015).

N-[(R)-1,2-dithiolane-3-pentanoyl]-L-glutamyl-l-alanine (CMX-2043) is an analogue of α-lipoic acid, an important mitochondrion-permeable antioxidant with its reduced-form dihydrolipoic acid (Zhang et al., 2016). CMX-2043 has been shown to be effective in reducing infarct damage in an animal model of myocardial IRI (Baguisi et al., 2016).

### Mitochondrial target: Biogenesis

As previously described, peroxisome proliferator-activated receptors (PPARs) are ligand-regulated transcription factors involved in mitochondrial biogenesis and the isoform PPARα with PGC-1α acts in the transcriptional regulation of myocardial glucose and lipid metabolism (Duncan, 2011). As already explained, after oxygen depletion, mitochondrial biogenesis is reduced after the suppression of PGC-1α by HIF (Thomas and Ashcroft, 2019). PPARs have been studied in order to understand their potential effects on restoring physiological mitochondrial biogenesis: for example, fibrates like Bezafibrate have been shown positive results in upregulating biogenesis *in vivo* (Viscomi et al., 2011), in ameliorating cardiovascular events incidence in STEMI patients in a randomized, controlled clinical trial (Madrid-Miller et al., 2010) and, in a longer clinical trial, Bezafibrate treatment was shown to reduce major cardiac events and mortality (Goldenberg et al., 2008; Goldenberg et al., 2009). Other drugs and compounds specific for PPARγ are Thiazolidinediones (TZDs): a class of hypoglycemic drugs for type 2 diabetes (Lalloyer and Staels, 2010). In this regard, Pioglitazone has been shown to have cardioprotective effects in several clinical studies. In a systematic review it has been proposed that PPARγ agonists probably lead to a reduction of recurrent stroke and total events of cardiovascular death, non-fatal myocardial infarction or non-fatal stroke (Liu and Wang, 2017). To the contrary, Rosiglitazone was shown not to be effective in swine and rat heart after I/R (Palee et al., 2011) and in clinical trials (Nissen and Wolski, 2007).

Another class of activators of mitochondrial biogenesis with cardioprotective effects against IRI are AMPK agonists, which act through activation of PGC-1α, and among them 5-aminoimidazole-4-carboxamide ribonucleoside (AICAR) (Viscomi et al., 2011). This compound activates AMPK, inducing genes, e.g. SIRT1, PGC-1α, and Parkin, leading to protection of neonatal rat cardiac myocytes from ischemic insult (Sunaga et al., 2019). In addition, in a mice model of sudden cardiac arrest, treatment with AICAR and metformin, another AMPK activator, preserved cardiac function from IRI. (*) Metformin, in addition to its effect on AMPK signaling pathway, was shown to have biphasic effects on mitochondria: at low concentrations it affects on oxygen consumption rate (OCR) and mitochondrial biogenesis of cardiomyocytes and at high concentrations in isolated human cardiac mitochondria it attenuates superoxide production and it inhibits Ca^2+^-induced mPTP opening (Emelyanova et al., 2021). This hypoglycemic drug has been shown to have cardioprotective effects in murine cardiac transplantation models limiting acute IRI (Chin et al., 2011) and, in rats undergoing cardiac I/R, metformin treatment ameliorates overall cardiac function after the reduction of mitochondrial fission, ROS production, apoptosis and mitochondrial swelling (Palee et al., 2020). Summarizing, even if several preclinical and clinical studies have suggested cardioprotective effects of metformin treatment (Holman et al., 2008; Mellbin et al., 2008), others have shown no beneficial long-term effects of this drug (Hartman et al., 2017).

### Mitochondrial target: Fission process

In cardiac I/R conditions mitochondrial fission, regulated by Drp1, has been associated with left ventricular (LV) impairment. In fact, Drp1 inhibitor Mdivi-1 avoids impairments of mitochondrial morphology and it reduces cytosolic Ca^2+^, preventing cell death and ameliorating cardiac function both *in vitro* and *ex vivo* (Sharp et al., 2014; Ong et al., 2010). Indeed, pre-ischemic administration of Mdivi-1 agent to mice efficiently inhibits the translocation of Drp1 to mitochondria, it reduces troponin I levels and the activity of lactate dehydrogenase (Ding et al., 2017), as well as it blocks mPTP opening, it attenuates apoptosis and it ameliorates mitochondrial function (Yu et al., 2017). The potential selective effect of Mdivi-1 during I/R, however, is still debated. Some studies suggest that Mdivi-1 has indirect effect in inhibiting Drp1 activity or in reducing mitochondrial segregation. In addition, its administration during reperfusion has been shown to exacerbate HL-1 cardiomyocytes apoptosis (Dong et al., 2016), as well as it has failed in decreasing infarct size in acute myocardial infarction of swine model (Ong et al., 2019).

Another class of more potent and specific Drp1 inhibitors are Drpitor1 and Drpitor1a, which blocked the GTPase activity of Drp1, in fact, Drpitor1a had a strong cardioprotective effect on I/R rodent model by inhibiting mitochondrial ROS production and mitochondrial fission. These compounds exerted a cardioprotective effect in the right ventricular rat cardiomyocytes (Wu et al., 2020). Future studies will provide additional insights into the potential benefits of Drpitor1 and Drpitor1a on attenuating I/R injury through the reduction of Ca^2+^ overload.

Drp1 activity might be inhibited also by a small peptide named P110 that blocked the interaction of fission proteins Fis1/Drp1. The effect of this peptide has been tested in different rat models of I/R, including primary cardiomyocytes, *ex vivo* heart model, and an *in vivo* myocardial infarction model, showing protective results. In addition, acute inhibition of fission immediately after reperfusion provided also long-term advantages up to 3 weeks after myocardial infarction. These findings deeply connect mitochondrial fragmentation to the reperfusion injury (Disatnik et al., 2013).

Recently, these findings have been further confirmed using hydralazine, which has cardioprotective effects through the repression of Drp1-mediated fission and reduces the infarct size in mice I/R model (Kalkhoran et al., 2022).

miR-499 is highly expressed in normal heart and its expression is reduced after ischemic events both *in vitro* and *ex vivo*. An antagomir targeting miR-499 has led to increased apoptosis, myocardial infarct size and decreased cardiac function. Interestingly, miR-499 is able to regulate apoptotic pathway acting on calcineurin-mediated dephosphorylation of Drp1, controlling Drp1-mediated initiation of the mitochondrial fission program with the transcriptional regulation of p53 (Wang et al., 2011).

In addition to fission inhibitors, the administration of a pharmacological mitochondrial fusion stimulator such as M1, has shown to have a significant cardioprotective effect before the onset of ischemia compared with what occurring at the initiation of reperfusion. M1 regulates the mitochondrial dynamics and attenuates mitochondrial damage by avoiding the reduction of fusion proteins and cardiac apoptosis during myocardial I/R (Maneechote et al., 2019).

### Target: Mitophagic process

Rapamycin is an inducer of mitophagic process thanks to the inhibition of mTORC1 pathway. This compound has positive effects on cardiomyocyte apoptosis and on infarct size (Gao et al., 2020). In addition, mTORC1 inhibition reduces adverse LV remodeling after myocardial infarction (Buss et al., 2009). mTOR comprises two structurally distinct multiprotein complexes, mTOR complexes one and 2 (mTORC1 and mTORC2). Thanks to pharmacological and molecular approaches specific to suppress one or two signaling, it has been revealed that in conditions of ischemic damage only preferentially mTORC2 signaling, given by inhibition of mTORC1, has cardioprotective role (Volkers et al., 2013).

Melatonin is a known hormone with antioxidant functions that has been deeply correlated to neuroprotection and cardioprotection from IRI. This compound inhibits mPTP opening (Andrabi et al., 2004) thanks to the preservation of mitochondrial cardiolipin integrity (Petrosillo et al., 2009). The protection is due to the fact that cardiolipin peroxidation leads to cytochrome c release and the consequent initiation of the apoptotic cascade. Moreover peroxidized cardiolipin has been previously recognized as an inducer of the mPTP (Petrosillo et al., 2006). Recently, another pathway has been recognized to be important for the cardioprotective effects of melatonin. These effects, both *in vitro* and *ex vivo,* are linked to inhibition of autophagy *via* AMPK/mTOR signaling and the AMPK activator AICAR or the mTOR inhibitor rapamycin counteract the positive effect of melatonin on IRI (Chen et al., 2018).

Erythropoietin (EPO) has obtained great interest as a therapeutic molecule for the treatment of IRI, but, due to its effects on the activation of erythropoiesis and thrombogenesis, the clinical results have not been so promising. Thus, a pyroglutamate helix B surface peptide (pHBP) has been developed in order to maintain tissue protective actions of EPO in the absence of its binding to the receptor (Ahmet et al., 2011). subsequent studies on the molecular mechanisms at the basis of the protective effects of this compound showed inhibition of the autophagy-related proteins (LC3II/LC3I) expression and enhanced expression of p-PI3K, p-Akt, and p-mTOR that leads to apoptosis reduction following IRI (Lin et al., 2020).

### Target: Mitochondrial functionality

In the recent years, since mitochondria have been shown to have a key role in controlling several pathways during the ischemic and reperfusion time, a very innovative approach to reduce tissue damage after I/R has been developed based on mitochondria transplantation. This procedure consists in the isolation of autologous mitochondria from a distant tissue uninfluenced by ischemia and in the transplantation of these “healthy” mitochondria in the cardiac site of ischemia right before reperfusion. These experiments have been performed *in vitro* and *in vivo* on rabbits using *in situ* blood-perfused heart and revealed a significant decrease in myocardial necrosis and a boost in postischemic function. The transplanted mitochondria maintained their function enhancing OCR and high-energy synthesis and were internalized in the tissue and cells 2 h after transplantation (Masuzawa et al., 2013). Consequent studies investigated different ways to deliver mitochondria to the injured heart. Thanks to labeled mitochondria and positron emission tomography, microcomputed tomography and magnetic resonance imaging it has been confirmed that perfusion through the coronary arteries is an efficient way of delivery of mitochondria (Cowan et al., 2016). A recent systematic review gathers all the preclinical and clinical studies on this technique. Twenty animal studies and two human studies showed a beneficial effect of mitochondrial transplantation in the treatment of IRI but further evidences are needed to understand the molecular mechanism and the potential side-effects in patients (Hayashida et al., 2021). The human studies were reported by the McCully’s group. Autologous mitochondrial transplantation was performed in pediatric patients connected to extracorporeal membrane oxygenation and the results are in support for I/R–associated myocardial dysfunction after cardiac surgical procedure. To specify, up to now these patients are the only ones eligible for mitochondrial autotransplantation. In another study, healthy autologous mitochondria harvested from nonischemic skeletal muscle were safely injected into damaged myocardium after ischemic injury for improvement in ventricular function (Emani et al., 2017). Recently, the group of Guariento published the results of a pilot study on 24 pediatric patients suggesting that this procedure enhances ventricular strain and it is relatively free of relevant side-effects, because it does not trigger arrhythmias, nor immune or inflammatory response (Guariento et al., 2021). Further investigations, however, will confirm the safety and the efficacy of this treatment, also defining the best delivery methods and possible long-term side effects. These first data, however, are promising and highlight the importance of mitochondria in the pathophysiology of myocardium. For example, the combination of SS31, the potent peptide with antioxidant properties previously described, and mitochondrial transplantation was tested *in vitro* and in a rodent model of acute myocardial IRI. This combined therapy is superior to either therapy alone in improving cardiac performance after IRI, paving the way to several combinations of mitochondria-based approaches (Lee et al., 2018).

## Conclusion

Since IHDs are one of the leading cause of mortality worldwide, in the last years the scientific community has made special efforts to study the pathological mechanisms underlying IRI. In this context, mitochondria hold a key position in controlling life and death of cardiomyocytes: dysfunctions in mitochondrial communication or interaction with other organelles lead to impaired Ca^2+^ signaling, mPTP opening, accumulation of mtROS and release of pro-apoptotic factors. Mitochondrial quality control requires a fine tuning of processes of fission, fusion, biogenesis and mitophagy, fundamental for a correct cellular functioning. During IRI, an out of control balance overcomes, leading to the final result of cell death and myocardial damage. Thus a variety of mitochondria-targeting therapies controlling the cellular/molecular processes involved in ischemia or reperfusion has been developed. Some of these therapeutic approaches have provided promising results in reducing cardiac damage in preclinical studies and this represents the basis for the development of well-designed clinical studies in patients with IRI.

## Fundings

This manuscript was funded by Maria Cecilia Hospital, GVM Care and Research, 48033 Cotignola, Italy. MRW was supported by the National Science Centre, Poland (grant UMO-2018/29/B/NZ1/00589).

## References

[B1] Abegunde D. O. Mathers C. D. Adam T. Ortegon M. Strong K. (2007). The burden and costs of chronic diseases in low-income and middle-income countries. Lancet 370 (9603), 1929–1938. 10.1016/S0140-6736(07)61696-1 18063029

[B2] Adlam V. J. Harrison J. C. Porteous C. M. James A. M. Smith R. A. Murphy M. P. (2005). Targeting an antioxidant to mitochondria decreases cardiac ischemia-reperfusion injury. FASEB J. 19 (9), 1088–1095. 10.1096/fj.05-3718com 15985532

[B3] Ahmet I. Tae H. J. Juhaszova M. Riordon D. R. Boheler K. R. Sollott S. J. (2011). A small nonerythropoietic helix B surface peptide based upon erythropoietin structure is cardioprotective against ischemic myocardial damage. Mol. Med. 17 (3-4), 194–200. 10.2119/molmed.2010.00235 21170473 PMC3060982

[B4] Alavian K. N. Beutner G. Lazrove E. Sacchetti S. Park H. A. Licznerski P. (2014). An uncoupling channel within the c-subunit ring of the F1FO ATP synthase is the mitochondrial permeability transition pore. Proc. Natl. Acad. Sci. U. S. A. 111 (29), 10580–10585. 10.1073/pnas.1401591111 24979777 PMC4115574

[B5] Allen M. E. Pennington E. R. Perry J. B. Dadoo S. Makrecka-Kuka M. Dambrova M. (2020). The cardiolipin-binding peptide elamipretide mitigates fragmentation of cristae networks following cardiac ischemia reperfusion in rats. Commun. Biol. 3 (1), 389. 10.1038/s42003-020-1101-3 32680996 PMC7368046

[B6] Ambekar C. S. Lee J. S. Cheung B. M. Chan L. C. Liang R. Kumana C. R. (2004). Chloramphenicol succinate, a competitive substrate and inhibitor of succinate dehydrogenase: Possible reason for its toxicity. Toxicol. Vitro 18 (4), 441–447. 10.1016/j.tiv.2003.12.010 15130601

[B7] Andrabi S. A. Sayeed I. Siemen D. Wolf G. Horn T. F. (2004). Direct inhibition of the mitochondrial permeability transition pore: A possible mechanism responsible for anti-apoptotic effects of melatonin. FASEB J. 18 (7), 869–871. 10.1096/fj.03-1031fje 15033929

[B8] Angebault C. Panel M. Lacote M. Rieusset J. Lacampagne A. Fauconnier J. (2020). Metformin reverses the enhanced myocardial SR/ER-Mitochondria interaction and impaired complex I-driven respiration in dystrophin-deficient mice. Front. Cell Dev. Biol. 8, 609493. 10.3389/fcell.2020.609493 33569379 PMC7868535

[B9] Antoniel M. Jones K. Antonucci S. Spolaore B. Fogolari F. Petronilli V. (2018). The unique histidine in OSCP subunit of F-ATP synthase mediates inhibition of the permeability transition pore by acidic pH. EMBO Rep. 19 (2), 257–268. 10.15252/embr.201744705 29217657 PMC5797955

[B10] Araszkiewicz A. Grygier M. Lesiak M. Grajek S. (2013). The impact of ischemia-reperfusion injury on the effectiveness of primary angioplasty in ST-segment elevation myocardial infarction. Postepy Kardiol. Interwencyjnej 9 (3), 275–281. 10.5114/pwki.2013.37509 24570732 PMC3915986

[B11] Azarashvili T. Odinokova I. Bakunts A. Ternovsky V. Krestinina O. Tyynela J. (2014). Potential role of subunit c of F0F1-ATPase and subunit c of storage body in the mitochondrial permeability transition. Effect of the phosphorylation status of subunit c on pore opening. Cell Calcium 55 (2), 69–77. 10.1016/j.ceca.2013.12.002 24380588

[B12] Baguisi A. Casale R. A. Kates S. A. Lader A. S. Stewart K. Beeuwkes R. 3rd (2016). CMX-2043 efficacy in a rat model of cardiac ischemia-reperfusion injury. J. Cardiovasc. Pharmacol. Ther. 21 (6), 563–569. 10.1177/1074248416640118 27113210

[B13] Baines C. P. Kaiser R. A. Purcell N. H. Blair N. S. Osinska H. Hambleton M. A. (2005). Loss of cyclophilin D reveals a critical role for mitochondrial permeability transition in cell death. Nature 434 (7033), 658–662. 10.1038/nature03434 15800627

[B14] Barzda V. Greenhalgh C. Aus der Au J. Elmore S. van Beek J. Squier J. (2005). Visualization of mitochondria in cardiomyocytes by simultaneous harmonic generation and fluorescence microscopy. Opt. Express 13 (20), 8263–8276. 10.1364/opex.13.008263 19498856

[B15] Bates E. Bode C. Costa M. Gibson C. M. Granger C. Direct Inhibition of delta-Protein Kinase C Enzyme to Limit Total Infarct Size in Acute Myocardial Infarction (DELTA MI) Investigators, (2008). Intracoronary KAI-9803 as an adjunct to primary percutaneous coronary intervention for acute ST-segment elevation myocardial infarction. Circulation 117 (7), 886–896. 10.1161/CIRCULATIONAHA.107.759167 18250271

[B16] Bers D. M. (2008). Calcium cycling and signaling in cardiac myocytes. Annu. Rev. Physiol. 70, 23–49. 10.1146/annurev.physiol.70.113006.100455 17988210

[B17] Billia F. Hauck L. Konecny F. Rao V. Shen J. Mak T. W. (2011). PTEN-inducible kinase 1 (PINK1)/Park6 is indispensable for normal heart function. Proc. Natl. Acad. Sci. U. S. A. 108 (23), 9572–9577. 10.1073/pnas.1106291108 21606348 PMC3111326

[B18] Birk A. V. Liu S. Soong Y. Mills W. Singh P. Warren J. D. (2013). The mitochondrial-targeted compound SS-31 re-energizes ischemic mitochondria by interacting with cardiolipin. J. Am. Soc. Nephrol. 24 (8), 1250–1261. 10.1681/ASN.2012121216 23813215 PMC3736700

[B19] Boncompagni S. Rossi A. E. Micaroni M. Beznoussenko G. V. Polishchuk R. S. Dirksen R. T. (2009). Mitochondria are linked to calcium stores in striated muscle by developmentally regulated tethering structures. Mol. Biol. Cell 20 (3), 1058–1067. 10.1091/mbc.E08-07-0783 19037102 PMC2633377

[B20] Bonora M. Bononi A. De Marchi E. Giorgi C. Lebiedzinska M. Marchi S. (2013). Role of the c subunit of the FO ATP synthase in mitochondrial permeability transition. Cell Cycle 12 (4), 674–683. 10.4161/cc.23599 23343770 PMC3594268

[B21] Bonora M. Giorgi C. Pinton P. (2022). Molecular mechanisms and consequences of mitochondrial permeability transition. Nat. Rev. Mol. Cell Biol. 23 (4), 266–285. 10.1038/s41580-021-00433-y 34880425

[B22] Bonora M. Morganti C. Morciano G. Pedriali G. Lebiedzinska-Arciszewska M. Aquila G. (2017). Mitochondrial permeability transition involves dissociation of F1FO ATP synthase dimers and C-ring conformation. EMBO Rep. 18 (7), 1077–1089. 10.15252/embr.201643602 28566520 PMC5494524

[B23] Bonora M. Patergnani S. Ramaccini D. Morciano G. Pedriali G. Kahsay A. E. (2020). Physiopathology of the permeability transition pore: Molecular mechanisms in human pathology. Biomolecules 10 (7), 998. 10.3390/biom10070998 32635556 PMC7408088

[B24] Bonora M. Wieckowski M. R. Sinclair D. A. Kroemer G. Pinton P. Galluzzi L. (2019). Targeting mitochondria for cardiovascular disorders: Therapeutic potential and obstacles. Nat. Rev. Cardiol. 16 (1), 33–55. 10.1038/s41569-018-0074-0 30177752 PMC6349394

[B25] Bouhamida E. Morciano G. Perrone M. Kahsay A. E. Della Sala M. Wieckowski M. R. (2022). The interplay of hypoxia signaling on mitochondrial dysfunction and inflammation in cardiovascular diseases and cancer: From molecular mechanisms to therapeutic approaches. Biol. (Basel) 11 (2), 300. 10.3390/biology11020300 PMC8869508 35205167

[B26] Brady N. R. Hamacher-Brady A. Gottlieb R. A. (2006). Proapoptotic BCL-2 family members and mitochondrial dysfunction during ischemia/reperfusion injury, a study employing cardiac HL-1 cells and GFP biosensors. Biochim. Biophys. Acta 1757 (5-6), 667–678. 10.1016/j.bbabio.2006.04.011 16730326

[B27] Brand M. D. Goncalves R. L. Orr A. L. Vargas L. Gerencser A. A. Borch Jensen M. (2016). Suppressors of superoxide-H2O2 production at site IQ of mitochondrial complex I protect against stem cell hyperplasia and ischemia-reperfusion injury. Cell Metab. 24 (4), 582–592. 10.1016/j.cmet.2016.08.012 27667666 PMC5061631

[B28] Burton R. A. B. Rog-Zielinska E. A. Corbett A. D. Peyronnet R. Bodi I. Fink M. (2017). Caveolae in rabbit ventricular myocytes: Distribution and dynamic diminution after cell isolation. Biophys. J. 113 (5), 1047–1059. 10.1016/j.bpj.2017.07.026 28877488 PMC5653872

[B29] Buss S. J. Muenz S. Riffel J. H. Malekar P. Hagenmueller M. Weiss C. S. (2009). Beneficial effects of Mammalian target of rapamycin inhibition on left ventricular remodeling after myocardial infarction. J. Am. Coll. Cardiol. 54 (25), 2435–2446. 10.1016/j.jacc.2009.08.031 20082935

[B30] Campo G. Morciano G. Pavasini R. Bonora M. Sbano L. Biscaglia S. (2016). Fo ATP synthase C subunit serum levels in patients with ST-segment Elevation Myocardial Infarction: Preliminary findings. Int. J. Cardiol. 221, 993–997. 10.1016/j.ijcard.2016.07.125 27441480

[B31] Cao J. L. Adaniya S. M. Cypress M. W. Suzuki Y. Kusakari Y. Jhun B. S. (2019). Role of mitochondrial Ca(2+) homeostasis in cardiac muscles. Arch. Biochem. Biophys. 663, 276–287. 10.1016/j.abb.2019.01.027 30684463 PMC6469710

[B32] Carrer A. Tommasin L. Sileikyte J. Ciscato F. Filadi R. Urbani A. (2021). Defining the molecular mechanisms of the mitochondrial permeability transition through genetic manipulation of F-ATP synthase. Nat. Commun. 12 (1), 4835. 10.1038/s41467-021-25161-x 34376679 PMC8355262

[B33] Chang D. T. Reynolds I. J. (2006). Mitochondrial trafficking and morphology in healthy and injured neurons. Prog. Neurobiol. 80 (5), 241–268. 10.1016/j.pneurobio.2006.09.003 17188795

[B34] Chatzianastasiou A. Bibli S. I. Andreadou I. Efentakis P. Kaludercic N. Wood M. E. (2016). Cardioprotection by H2S donors: Nitric oxide-dependent and independent mechanisms. J. Pharmacol. Exp. Ther. 358 (3), 431–440. 10.1124/jpet.116.235119 27342567 PMC6047225

[B35] Chen Q. Lesnefsky E. J. (2011). Blockade of electron transport during ischemia preserves bcl-2 and inhibits opening of the mitochondrial permeability transition pore. FEBS Lett. 585 (6), 921–926. 10.1016/j.febslet.2011.02.029 21354418 PMC3076511

[B36] Chen Q. Moghaddas S. Hoppel C. L. Lesnefsky E. J. (2008). Ischemic defects in the electron transport chain increase the production of reactive oxygen species from isolated rat heart mitochondria. Am. J. Physiol. Cell Physiol. 294 (2), C460–C466. 10.1152/ajpcell.00211.2007 18077608

[B37] Chen Q. Moghaddas S. Hoppel C. L. Lesnefsky E. J. (2006). Reversible blockade of electron transport during ischemia protects mitochondria and decreases myocardial injury following reperfusion. J. Pharmacol. Exp. Ther. 319 (3), 1405–1412. 10.1124/jpet.106.110262 16990510

[B38] Chen W. R. Liu H. B. Chen Y. D. Sha Y. Ma Q. Zhu P. J. (2018). Melatonin attenuates myocardial ischemia/reperfusion injury by inhibiting autophagy via an AMPK/mTOR signaling pathway. Cell. Physiol. biochem. 47 (5), 2067–2076. 10.1159/000491474 29975938

[B39] Chen Y. Csordas G. Jowdy C. Schneider T. G. Csordas N. Wang W. (2012). Mitofusin 2-containing mitochondrial-reticular microdomains direct rapid cardiomyocyte bioenergetic responses via interorganelle Ca(2+) crosstalk. Circ. Res. 111 (7), 863–875. 10.1161/CIRCRESAHA.112.266585 22777004 PMC3444672

[B40] Chen Y. Dorn G. W. (2013). PINK1-phosphorylated mitofusin 2 is a Parkin receptor for culling damaged mitochondria. Science 340 (6131), 471–475. 10.1126/science.1231031 23620051 PMC3774525

[B41] Chen Y. Liu C. Li J. Zhou P. Zhao X. Chen R. (2021). LATS2 deletion attenuates myocardial ischemia-reperfusion injury by promoting mitochondrial biogenesis. Oxid. Med. Cell. Longev. 2021, 1058872. 10.1155/2021/1058872 34457109 PMC8390173

[B42] Cheng D. Zheng J. Hu F. Lv W. Lu C. (2021). Abnormal mitochondria-endoplasmic reticulum communication promotes myocardial infarction. Front. Physiol. 12, 717187. 10.3389/fphys.2021.717187 34413791 PMC8369510

[B43] Cheng Y. Liu D. Z. Zhang C. X. Cui H. Liu M. Zhang B. L. (2019). Mitochondria-targeted antioxidant delivery for precise treatment of myocardial ischemia-reperfusion injury through a multistage continuous targeted strategy. Nanomedicine 16, 236–249. 10.1016/j.nano.2018.12.014 30639669

[B44] Chin J. T. Troke J. J. Kimura N. Itoh S. Wang X. Palmer O. P. (2011). A novel cardioprotective agent in cardiac transplantation: Metformin activation of AMP-activated protein kinase decreases acute ischemia-reperfusion injury and chronic rejection. Yale J. Biol. Med. 84 (4), 423–432. 22180679 PMC3238328

[B45] Chouchani E. T. Methner C. Nadtochiy S. M. Logan A. Pell V. R. Ding S. (2013). Cardioprotection by S-nitrosation of a cysteine switch on mitochondrial complex I. Nat. Med. 19 (6), 753–759. 10.1038/nm.3212 23708290 PMC4019998

[B46] Chouchani E. T. Pell V. R. Gaude E. Aksentijevic D. Sundier S. Y. Robb E. L. (2014). Ischaemic accumulation of succinate controls reperfusion injury through mitochondrial ROS. Nature 515 (7527), 431–435. 10.1038/nature13909 25383517 PMC4255242

[B47] Chouchani E. T. Pell V. R. James A. M. Work L. M. Saeb-Parsy K. Frezza C. (2016). A unifying mechanism for mitochondrial superoxide production during ischemia-reperfusion injury. Cell Metab. 23 (2), 254–263. 10.1016/j.cmet.2015.12.009 26777689

[B48] Choudhury S. Bae S. Ke Q. Lee J. Y. Kim J. Kang P. M. (2011). Mitochondria to nucleus translocation of AIF in mice lacking Hsp70 during ischemia/reperfusion. Basic Res. Cardiol. 106 (3), 397–407. 10.1007/s00395-011-0164-1 21387140 PMC3205442

[B49] Churchill E. N. Szweda L. I. (2005). Translocation of deltaPKC to mitochondria during cardiac reperfusion enhances superoxide anion production and induces loss in mitochondrial function. Arch. Biochem. Biophys. 439 (2), 194–199. 10.1016/j.abb.2005.05.007 15963450

[B50] Cohen M. M. Tareste D. (2018). Recent insights into the structure and function of Mitofusins in mitochondrial fusion. F1000Res 7, F1000Res. 10.12688/f1000research.16629.1 PMC6317495 30647902

[B51] Cowan D. B. Yao R. Akurathi V. Snay E. R. Thedsanamoorthy J. K. Zurakowski D. (2016). Intracoronary delivery of mitochondria to the ischemic heart for cardioprotection. PLoS One 11 (8), e0160889. 10.1371/journal.pone.0160889 27500955 PMC4976938

[B52] Cung T. T. Morel O. Cayla G. Rioufol G. Garcia-Dorado D. Angoulvant D. (2015). Cyclosporine before PCI in patients with acute myocardial infarction. N. Engl. J. Med. 373 (11), 1021–1031. 10.1056/NEJMoa1505489 26321103

[B53] Dare A. J. Logan A. Prime T. A. Rogatti S. Goddard M. Bolton E. M. (2015). The mitochondria-targeted anti-oxidant MitoQ decreases ischemia-reperfusion injury in a murine syngeneic heart transplant model. J. Heart Lung Transpl. 34 (11), 1471–1480. 10.1016/j.healun.2015.05.007 PMC4626443 26140808

[B54] De la Fuente S. Sheu S. S. (2019). SR-mitochondria communication in adult cardiomyocytes: A close relationship where the Ca(2+) has a lot to say. Arch. Biochem. Biophys. 663, 259–268. 10.1016/j.abb.2019.01.026 30685253 PMC6377816

[B55] De Vos K. J. Morotz G. M. Stoica R. Tudor E. L. Lau K. F. Ackerley S. (2012). VAPB interacts with the mitochondrial protein PTPIP51 to regulate calcium homeostasis. Hum. Mol. Genet. 21 (6), 1299–1311. 10.1093/hmg/ddr559 22131369 PMC3284118

[B56] Denton R. M. McCormack J. G. (1990). Ca2+ as a second messenger within mitochondria of the heart and other tissues. Annu. Rev. Physiol. 52, 451–466. 10.1146/annurev.ph.52.030190.002315 2184763

[B57] Desai R. East D. A. Hardy L. Faccenda D. Rigon M. Crosby J. (2020). Mitochondria form contact sites with the nucleus to couple prosurvival retrograde response. Sci. Adv. 6 (51), eabc9955. 10.1126/sciadv.abc9955 33355129 PMC11206220

[B58] Ding M. Dong Q. Liu Z. Liu Z. Qu Y. Li X. (2017). Inhibition of dynamin-related protein 1 protects against myocardial ischemia-reperfusion injury in diabetic mice. Cardiovasc. Diabetol. 16 (1), 19. 10.1186/s12933-017-0501-2 28173848 PMC5297196

[B59] Ding Q. Qi Y. Tsang S. Y. (2021). Mitochondrial biogenesis, mitochondrial dynamics, and mitophagy in the maturation of cardiomyocytes. Cells 10 (9), 2463. 10.3390/cells10092463 34572112 PMC8466139

[B60] Disatnik M. H. Ferreira J. C. Campos J. C. Gomes K. S. Dourado P. M. Qi X. (2013). Acute inhibition of excessive mitochondrial fission after myocardial infarction prevents long-term cardiac dysfunction. J. Am. Heart Assoc. 2 (5), e000461. 10.1161/JAHA.113.000461 24103571 PMC3835263

[B61] Diwan A. Krenz M. Syed F. M. Wansapura J. Ren X. Koesters A. G. (2007). Inhibition of ischemic cardiomyocyte apoptosis through targeted ablation of Bnip3 restrains postinfarction remodeling in mice. J. Clin. Invest. 117 (10), 2825–2833. 10.1172/JCI32490 17909626 PMC1994631

[B62] Dong Y. Undyala V. V. R. Przyklenk K. (2016). Inhibition of mitochondrial fission as a molecular target for cardioprotection: Critical importance of the timing of treatment. Basic Res. Cardiol. 111 (5), 59. 10.1007/s00395-016-0578-x 27573530

[B63] Dorn G. W. 2nd Vega R. B. Kelly D. P. (2015). Mitochondrial biogenesis and dynamics in the developing and diseased heart. Genes Dev. 29 (19), 1981–1991. 10.1101/gad.269894.115 26443844 PMC4604339

[B64] Dorner A. Lynetskiy O. Euler G. Landmesser U. Schluter K. D. Heger J. (2021). Mitochondria isolated from hearts subjected to ischemia/reperfusion benefit from adenine nucleotide translocase 1 overexpression. Membr. (Basel) 11 (11), 836. 10.3390/membranes11110836 PMC8619488 34832065

[B65] Duncan J. G. (2011). Peroxisome proliferator activated receptor-alpha (PPARα) and PPAR gamma coactivator-1alpha (PGC-1α) regulation of cardiac metabolism in diabetes. Pediatr. Cardiol. 32 (3), 323–328. 10.1007/s00246-011-9889-8 21286700 PMC3143064

[B66] Dzeja P. P. Bortolon R. Perez-Terzic C. Holmuhamedov E. L. Terzic A. (2002). Energetic communication between mitochondria and nucleus directed by catalyzed phosphotransfer. Proc. Natl. Acad. Sci. U. S. A. 99 (15), 10156–10161. 10.1073/pnas.152259999 12119406 PMC126640

[B67] Emani S. M. Piekarski B. L. Harrild D. Del Nido P. J. McCully J. D. (2017). Autologous mitochondrial transplantation for dysfunction after ischemia-reperfusion injury. J. Thorac. Cardiovasc. Surg. 154 (1), 286–289. 10.1016/j.jtcvs.2017.02.018 28283239

[B68] Emelyanova L. Bai X. Yan Y. Bosnjak Z. J. Kress D. Warner C. (2021). Biphasic effect of metformin on human cardiac energetics. Transl. Res. 229, 5–23. 10.1016/j.trsl.2020.10.002 33045408 PMC10655614

[B69] Fabiato A. (1983). Calcium-induced release of calcium from the cardiac sarcoplasmic reticulum. Am. J. Physiol. 245 (1), C1–C14. 10.1152/ajpcell.1983.245.1.C1 6346892

[B70] Fabregat-Andres O. Paredes F. Monsalve M. Milara J. Ridocci-Soriano F. Gonzalez-Hervas S. (2016). mRNA PGC-1α levels in blood samples reliably correlates with its myocardial expression: Study in patients undergoing cardiac surgery. Anatol. J. Cardiol. 16 (8), 622–629. 10.5152/AnatolJCardiol.2015.6466 27004709 PMC5368522

[B71] Fabregat-Andres O. Ridocci-Soriano F. Estornell-Erill J. Corbi-Pascual M. Valle-Munoz A. Berenguer-Jofresa A. (2015). Blood PGC-1α concentration predicts myocardial salvage and ventricular remodeling after ST-segment elevation acute myocardial infarction. Rev. Esp. Cardiol. 68 (5), 408–416. 10.1016/j.rec.2014.05.020 25440044

[B72] Fabregat-Andres O. Tierrez A. Mata M. Estornell-Erill J. Ridocci-Soriano F. Monsalve M. (2011). Induction of PGC-1α expression can be detected in blood samples of patients with ST-segment elevation acute myocardial infarction. PLoS One 6 (11), e26913. 10.1371/journal.pone.0026913 22087236 PMC3210132

[B73] Fang J. Song X. W. Tian J. Chen H. Y. Li D. F. Wang J. F. (2012). Overexpression of microRNA-378 attenuates ischemia-induced apoptosis by inhibiting caspase-3 expression in cardiac myocytes. Apoptosis 17 (4), 410–423. 10.1007/s10495-011-0683-0 22119805

[B74] Fantinati A. Morciano G. Turrin G. Pedriali G. Pacifico S. Preti D. (2022). Identification of small-molecule urea derivatives as PTPC modulators targeting the c subunit of F1/Fo-ATP synthase. Bioorg. Med. Chem. Lett. 72, 128822. 10.1016/j.bmcl.2022.128822 35636649

[B75] Fernandez-Marcos P. J. Auwerx J. (2011). Regulation of PGC-1α, a nodal regulator of mitochondrial biogenesis. Am. J. Clin. Nutr. 93 (4), 884S–890S. 10.3945/ajcn.110.001917 21289221 PMC3057551

[B76] Filadi R. Pendin D. Pizzo P. (2018). Mitofusin 2: From functions to disease. Cell Death Dis. 9 (3), 330. 10.1038/s41419-017-0023-6 29491355 PMC5832425

[B77] Forbes M. S. Sperelakis N. (1982). Association between mitochondria and gap junctions in mammalian myocardial cells. Tissue Cell 14 (1), 25–37. 10.1016/0040-8166(82)90004-0 7089964

[B78] Forini F. Canale P. Nicolini G. Iervasi G. (2020). Mitochondria-targeted drug delivery in cardiovascular disease: A long road to nano-cardio medicine. Pharmaceutics 12 (11), 1122. 10.3390/pharmaceutics12111122 33233847 PMC7699942

[B79] Foster C. R. Satomi S. Kato Y. Patel H. H. (2020). The caveolar-mitochondrial interface: Regulation of cellular metabolism in physiology and pathophysiology. Biochem. Soc. Trans. 48 (1), 165–177. 10.1042/BST20190388 32010944

[B80] Frank A. Bonney M. Bonney S. Weitzel L. Koeppen M. Eckle T. (2012). Myocardial ischemia reperfusion injury: From basic science to clinical bedside. Semin. Cardiothorac. Vasc. Anesth. 16 (3), 123–132. 10.1177/1089253211436350 22368166 PMC3457795

[B81] Frey T. G. Mannella C. A. (2000). The internal structure of mitochondria. Trends biochem. Sci. 25 (7), 319–324. 10.1016/s0968-0004(00)01609-1 10871882

[B82] Fridolfsson H. N. Kawaraguchi Y. Ali S. S. Panneerselvam M. Niesman I. R. Finley J. C. (2012). Mitochondria-localized caveolin in adaptation to cellular stress and injury. FASEB J. 26 (11), 4637–4649. 10.1096/fj.12-215798 22859372 PMC4050367

[B83] Galluzzi L. Kepp O. Trojel-Hansen C. Kroemer G. (2012). Mitochondrial control of cellular life, stress, and death. Circ. Res. 111 (9), 1198–1207. 10.1161/CIRCRESAHA.112.268946 23065343

[B84] Gao G. Chen W. Yan M. Liu J. Luo H. Wang C. (2020). Rapamycin regulates the balance between cardiomyocyte apoptosis and autophagy in chronic heart failure by inhibiting mTOR signaling. Int. J. Mol. Med. 45 (1), 195–209. 10.3892/ijmm.2019.4407 31746373 PMC6889932

[B85] Garbern J. C. Lee R. T. (2021). Mitochondria and metabolic transitions in cardiomyocytes: Lessons from development for stem cell-derived cardiomyocytes. Stem Cell Res. Ther. 12 (1), 177. 10.1186/s13287-021-02252-6 33712058 PMC7953594

[B86] Garcia J. J. Morales-Rios E. Cortes-Hernandez P. Rodriguez-Zavala J. S. (2006). The inhibitor protein (IF1) promotes dimerization of the mitochondrial F1F0-ATP synthase. Biochemistry 45 (42), 12695–12703. 10.1021/bi060339j 17042487

[B87] Garesse R. Vallejo C. G. (2001). Animal mitochondrial biogenesis and function: A regulatory cross-talk between two genomes. Gene 263 (1-2), 1–16. 10.1016/s0378-1119(00)00582-5 11223238

[B88] Ghaffari S. Kazemi B. Toluey M. Sepehrvand N. (2013). The effect of prethrombolytic cyclosporine-A injection on clinical outcome of acute anterior ST-elevation myocardial infarction. Cardiovasc. Ther. 31 (4), e34–e39. 10.1111/1755-5922.12010 23061531

[B89] Gibson C. M. Giugliano R. P. Kloner R. A. Bode C. Tendera M. Janosi A. (2016). EMBRACE STEMI study: A phase 2a trial to evaluate the safety, tolerability, and efficacy of intravenous MTP-131 on reperfusion injury in patients undergoing primary percutaneous coronary intervention. Eur. Heart J. 37 (16), 1296–1303. 10.1093/eurheartj/ehv597 26586786

[B90] Giguere V. (2008). Transcriptional control of energy homeostasis by the estrogen-related receptors. Endocr. Rev. 29 (6), 677–696. 10.1210/er.2008-0017 18664618

[B91] Giorgi C. Marchi S. Pinton P. (2018). The machineries, regulation and cellular functions of mitochondrial calcium. Nat. Rev. Mol. Cell Biol. 19 (11), 713–730. 10.1038/s41580-018-0052-8 30143745

[B92] Giorgio V. Burchell V. Schiavone M. Bassot C. Minervini G. Petronilli V. (2017). Ca(2+) binding to F-ATP synthase beta subunit triggers the mitochondrial permeability transition. EMBO Rep. 18 (7), 1065–1076. 10.15252/embr.201643354 28507163 PMC5494526

[B93] Gleyzer N. Vercauteren K. Scarpulla R. C. (2005). Control of mitochondrial transcription specificity factors (TFB1M and TFB2M) by nuclear respiratory factors (NRF-1 and NRF-2) and PGC-1 family coactivators. Mol. Cell. Biol. 25 (4), 1354–1366. 10.1128/MCB.25.4.1354-1366.2005 15684387 PMC548005

[B94] Goldenberg I. Benderly M. Goldbourt U. Group B. I. P. S. (2008). Secondary prevention with bezafibrate therapy for the treatment of dyslipidemia: An extended follow-up of the BIP trial. J. Am. Coll. Cardiol. 51 (4), 459–465. 10.1016/j.jacc.2007.09.048 18222357

[B95] Goldenberg I. Boyko V. Tennenbaum A. Tanne D. Behar S. Guetta V. (2009). Long-term benefit of high-density lipoprotein cholesterol-raising therapy with bezafibrate: 16-year mortality follow-up of the bezafibrate infarction prevention trial. Arch. Intern. Med. 169 (5), 508–514. 10.1001/archinternmed.2008.584 19273782

[B96] Gomez L. Paillard M. Thibault H. Derumeaux G. Ovize M. (2008). Inhibition of GSK3beta by postconditioning is required to prevent opening of the mitochondrial permeability transition pore during reperfusion. Circulation 117 (21), 2761–2768. 10.1161/CIRCULATIONAHA.107.755066 18490522

[B97] Gomez L. Thiebaut P. A. Paillard M. Ducreux S. Abrial M. Crola Da Silva C. (2016). The SR/ER-mitochondria calcium crosstalk is regulated by GSK3β during reperfusion injury. Cell Death Differ. 23 (2), 313–322. 10.1038/cdd.2015.101 26206086 PMC4716295

[B98] Granger D. N. Kvietys P. R. (2015). Reperfusion injury and reactive oxygen species: The evolution of a concept. Redox Biol. 6, 524–551. 10.1016/j.redox.2015.08.020 26484802 PMC4625011

[B99] Griffiths E. J. Halestrap A. P. (1995). Mitochondrial non-specific pores remain closed during cardiac ischaemia, but open upon reperfusion. Biochem. J. 307 (1), 93–98. 10.1042/bj3070093 7717999 PMC1136749

[B100] Griparic L. van der Wel N. N. Orozco I. J. Peters P. J. van der Bliek A. M. (2004). Loss of the intermembrane space protein Mgm1/OPA1 induces swelling and localized constrictions along the lengths of mitochondria. J. Biol. Chem. 279 (18), 18792–18798. 10.1074/jbc.M400920200 14970223

[B101] Guan L. Che Z. Meng X. Yu Y. Li M. Yu Z. (2019). MCU Up-regulation contributes to myocardial ischemia-reperfusion Injury through calpain/OPA-1-mediated mitochondrial fusion/mitophagy Inhibition. J. Cell. Mol. Med. 23 (11), 7830–7843. 10.1111/jcmm.14662 31502361 PMC6815825

[B102] Guariento A. Piekarski B. L. Doulamis I. P. Blitzer D. Ferraro A. M. Harrild D. M. (2021). Autologous mitochondrial transplantation for cardiogenic shock in pediatric patients following ischemia-reperfusion injury. J. Thorac. Cardiovasc. Surg. 162 (3), 992–1001. 10.1016/j.jtcvs.2020.10.151 33349443

[B103] Guha M. Avadhani N. G. (2013). Mitochondrial retrograde signaling at the crossroads of tumor bioenergetics, genetics and epigenetics. Mitochondrion 13 (6), 577–591. 10.1016/j.mito.2013.08.007 24004957 PMC3832239

[B104] Halestrap A. P. Clarke S. J. Javadov S. A. (2004). Mitochondrial permeability transition pore opening during myocardial reperfusion-a target for cardioprotection. Cardiovasc. Res. 61 (3), 372–385. 10.1016/S0008-6363(03)00533-9 14962470

[B105] Halestrap A. P. Connern C. P. Griffiths E. J. Kerr P. M. (1997). Cyclosporin A binding to mitochondrial cyclophilin inhibits the permeability transition pore and protects hearts from ischaemia/reperfusion injury. Mol. Cell. Biochem. 174 (1-2), 167–172. 10.1023/a:1006879618176 9309682

[B106] Hall A. R. Burke N. Dongworth R. K. Hausenloy D. J. (2014). Mitochondrial fusion and fission proteins: Novel therapeutic targets for combating cardiovascular disease. Br. J. Pharmacol. 171 (8), 1890–1906. 10.1111/bph.12516 24328763 PMC3976611

[B107] Hall A. R. Burke N. Dongworth R. K. Kalkhoran S. B. Dyson A. Vicencio J. M. (2016). Hearts deficient in both Mfn1 and Mfn2 are protected against acute myocardial infarction. Cell Death Dis. 7, e2238. 10.1038/cddis.2016.139 27228353 PMC4917668

[B108] Ham P. B. 3rd Raju R. (2017). Mitochondrial function in hypoxic ischemic injury and influence of aging. Prog. Neurobiol. 157, 92–116. 10.1016/j.pneurobio.2016.06.006 27321753 PMC5161736

[B109] Hamacher-Brady A. Brady N. R. Logue S. E. Sayen M. R. Jinno M. Kirshenbaum L. A. (2007). Response to myocardial ischemia/reperfusion injury involves Bnip3 and autophagy. Cell Death Differ. 14 (1), 146–157. 10.1038/sj.cdd.4401936 16645637

[B110] Hartman M. H. T. Prins J. K. B. Schurer R. A. J. Lipsic E. Lexis C. P. H. van der Horst-Schrivers A. N. A. (2017). Two-year follow-up of 4 months metformin treatment vs. placebo in ST-elevation myocardial infarction: Data from the GIPS-III RCT. Clin. Res. Cardiol. 106 (12), 939–946. 10.1007/s00392-017-1140-z 28755285 PMC5696505

[B111] Hausenloy D. J. Schulz R. Girao H. Kwak B. R. De Stefani D. Rizzuto R. (2020). Mitochondrial ion channels as targets for cardioprotection. J. Cell. Mol. Med. 24 (13), 7102–7114. 10.1111/jcmm.15341 32490600 PMC7339171

[B112] Hausenloy D. J. Yellon D. M. (2013). Myocardial ischemia-reperfusion injury: A neglected therapeutic target. J. Clin. Invest. 123 (1), 92–100. 10.1172/JCI62874 23281415 PMC3533275

[B113] Hausenloy D. J. Yellon D. M. (2003). The mitochondrial permeability transition pore: Its fundamental role in mediating cell death during ischaemia and reperfusion. J. Mol. Cell. Cardiol. 35 (4), 339–341. 10.1016/s0022-2828(03)00043-9 12689812

[B114] Haworth R. A. Hunter D. R. (1979). The Ca2+-induced membrane transition in mitochondria. II. Nature of the Ca2+ trigger site. Arch. Biochem. Biophys. 195 (2), 460–467. 10.1016/0003-9861(79)90372-2 38751

[B115] Hayashi T. Martone M. E. Yu Z. Thor A. Doi M. Holst M. J. (2009). Three-dimensional electron microscopy reveals new details of membrane systems for Ca2+ signaling in the heart. J. Cell Sci. 122 (7), 1005–1013. 10.1242/jcs.028175 19295127 PMC2720931

[B116] Hayashida K. Takegawa R. Shoaib M. Aoki T. Choudhary R. C. Kuschner C. E. (2021). Mitochondrial transplantation therapy for ischemia reperfusion injury: A systematic review of animal and human studies. J. Transl. Med. 19 (1), 214. 10.1186/s12967-021-02878-3 34001191 PMC8130169

[B117] He X. Bi X. Y. Lu X. Z. Zhao M. Yu X. J. Sun L. (2015). Reduction of mitochondria-endoplasmic reticulum interactions by acetylcholine protects human umbilical vein endothelial cells from hypoxia/reoxygenation injury. Arterioscler. Thromb. Vasc. Biol. 35 (7), 1623–1634. 10.1161/ATVBAHA.115.305469 25977565

[B118] Heusch G. (2019). Myocardial ischemia: Lack of coronary blood flow, myocardial oxygen supply-demand imbalance, or what? Am. J. Physiol. Heart Circ. Physiol. 316 (6), H1439–H1446. 10.1152/ajpheart.00139.2019 31002282 PMC7137753

[B119] Hollander J. M. Thapa D. Shepherd D. L. (2014). Physiological and structural differences in spatially distinct subpopulations of cardiac mitochondria: Influence of cardiac pathologies. Am. J. Physiol. Heart Circ. Physiol. 307 (1), H1–H14. 10.1152/ajpheart.00747.2013 24778166 PMC4080170

[B120] Holman R. R. Paul S. K. Bethel M. A. Matthews D. R. Neil H. A. (2008). 10-year follow-up of intensive glucose control in type 2 diabetes. N. Engl. J. Med. 359 (15), 1577–1589. 10.1056/NEJMoa0806470 18784090

[B121] Hom J. Sheu S. S. (2009). Morphological dynamics of mitochondria-a special emphasis on cardiac muscle cells. J. Mol. Cell. Cardiol. 46 (6), 811–820. 10.1016/j.yjmcc.2009.02.023 19281816 PMC2995918

[B122] Honda T. Kaikita K. Tsujita K. Hayasaki T. Matsukawa M. Fuchigami S. (2008). Pioglitazone, a peroxisome proliferator-activated receptor-gamma agonist, attenuates myocardial ischemia-reperfusion injury in mice with metabolic disorders. J. Mol. Cell. Cardiol. 44 (5), 915–926. 10.1016/j.yjmcc.2008.03.004 18436235

[B123] Hoppins S. Lackner L. Nunnari J. (2007). The machines that divide and fuse mitochondria. Annu. Rev. Biochem. 76, 751–780. 10.1146/annurev.biochem.76.071905.090048 17362197

[B124] Hu Q. Zhang H. Gutierrez Cortes N. Wu D. Wang P. Zhang J. (2020). Increased Drp1 acetylation by lipid overload induces cardiomyocyte death and heart dysfunction. Circ. Res. 126 (4), 456–470. 10.1161/CIRCRESAHA.119.315252 31896304 PMC7035202

[B125] Huang G. Docampo R. (2020). The mitochondrial calcium uniporter interacts with subunit c of the ATP synthase of trypanosomes and humans. mBio 11 (2), e00268–20. 10.1128/mBio.00268-20 32184243 PMC7078472

[B126] Hurst S. Gonnot F. Dia M. Crola Da Silva C. Gomez L. Sheu S. S. (2020). Phosphorylation of cyclophilin D at serine 191 regulates mitochondrial permeability transition pore opening and cell death after ischemia-reperfusion. Cell Death Dis. 11 (8), 661. 10.1038/s41419-020-02864-5 32814770 PMC7438327

[B127] Ikeda G. Matoba T. Ishikita A. Nagaoka K. Nakano K. Koga J. I. (2021). Nanoparticle-mediated simultaneous targeting of mitochondrial injury and inflammation attenuates myocardial ischemia-reperfusion injury. J. Am. Heart Assoc. 10 (12), e019521. 10.1161/JAHA.120.019521 34056918 PMC8477875

[B128] Ikeda G. Matoba T. Nakano Y. Nagaoka K. Ishikita A. Nakano K. (2016). Nanoparticle-mediated targeting of cyclosporine A enhances cardioprotection against ischemia-reperfusion injury through inhibition of mitochondrial permeability transition pore opening. Sci. Rep. 6, 20467. 10.1038/srep20467 26861678 PMC4748220

[B129] Ikeda S. Kong S. W. Lu J. Bisping E. Zhang H. Allen P. D. (2007). Altered microRNA expression in human heart disease. Physiol. Genomics 31 (3), 367–373. 10.1152/physiolgenomics.00144.2007 17712037

[B130] Ikeda Y. Shirakabe A. Maejima Y. Zhai P. Sciarretta S. Toli J. (2015). Endogenous Drp1 mediates mitochondrial autophagy and protects the heart against energy stress. Circ. Res. 116 (2), 264–278. 10.1161/CIRCRESAHA.116.303356 25332205

[B131] Inagaki K. Chen L. Ikeno F. Lee F. H. Imahashi K. Bouley D. M. (2003). Inhibition of delta-protein kinase C protects against reperfusion injury of the ischemic heart *in vivo* . Circulation 108 (19), 2304–2307. 10.1161/01.CIR.0000101682.24138.36 14597593

[B132] Iwasawa R. Mahul-Mellier A. L. Datler C. Pazarentzos E. Grimm S. (2011). Fis1 and Bap31 bridge the mitochondria-ER interface to establish a platform for apoptosis induction. EMBO J. 30 (3), 556–568. 10.1038/emboj.2010.346 21183955 PMC3034017

[B133] Jin Q. Li R. Hu N. Xin T. Zhu P. Hu S. (2018). DUSP1 alleviates cardiac ischemia/reperfusion injury by suppressing the Mff-required mitochondrial fission and Bnip3-related mitophagy via the JNK pathways. Redox Biol. 14, 576–587. 10.1016/j.redox.2017.11.004 29149759 PMC5691221

[B134] Jin S. M. Lazarou M. Wang C. Kane L. A. Narendra D. P. Youle R. J. (2010). Mitochondrial membrane potential regulates PINK1 import and proteolytic destabilization by PARL. J. Cell Biol. 191 (5), 933–942. 10.1083/jcb.201008084 21115803 PMC2995166

[B135] Juhaszova M. Zorov D. B. Kim S. H. Pepe S. Fu Q. Fishbein K. W. (2004). Glycogen synthase kinase-3beta mediates convergence of protection signaling to inhibit the mitochondrial permeability transition pore. J. Clin. Invest. 113 (11), 1535–1549. 10.1172/JCI19906 15173880 PMC419483

[B136] Kalkhoran S. B. Kriston-Vizi J. Hernandez-Resendiz S. Crespo-Avilan G. E. Rosdah A. A. Lees J. G. (2022). Hydralazine protects the heart against acute ischaemia/reperfusion injury by inhibiting Drp1-mediated mitochondrial fission. Cardiovasc. Res. 118 (1), 282–294. 10.1093/cvr/cvaa343 33386841 PMC8752357

[B137] Kang P. M. Haunstetter A. Aoki H. Usheva A. Izumo S. UshevA A. (2000). Morphological and molecular characterization of adult cardiomyocyte apoptosis during hypoxia and reoxygenation. Circ. Res. 87 (2), 118–125. 10.1161/01.res.87.2.118 10903995

[B138] Karch J. Bround M. J. Khalil H. Sargent M. A. Latchman N. Terada N. (2019). Inhibition of mitochondrial permeability transition by deletion of the ANT family and CypD. Sci. Adv. 5 (8), eaaw4597. 10.1126/sciadv.aaw4597 31489369 PMC6713508

[B139] Karch J. Molkentin J. D. (2014). Identifying the components of the elusive mitochondrial permeability transition pore. Proc. Natl. Acad. Sci. U. S. A. 111 (29), 10396–10397. 10.1073/pnas.1410104111 25002521 PMC4115577

[B140] Karlsson L. O. Bergh N. Grip L. (2012). Cyclosporine A, 2.5 mg/kg, does not reduce myocardial infarct size in a porcine model of ischemia and reperfusion. J. Cardiovasc. Pharmacol. Ther. 17 (2), 159–163. 10.1177/1074248411407636 21572075

[B141] Karlsson L. O. Zhou A. X. Larsson E. Astrom-Olsson K. Mansson C. Akyurek L. M. (2010). Cyclosporine does not reduce myocardial infarct size in a porcine ischemia-reperfusion model. J. Cardiovasc. Pharmacol. Ther. 15 (2), 182–189. 10.1177/1074248410362074 20435992

[B142] Karwi Q. G. Bornbaum J. Boengler K. Torregrossa R. Whiteman M. Wood M. E. (2017). AP39, a mitochondria-targeting hydrogen sulfide (H2 S) donor, protects against myocardial reperfusion injury independently of salvage kinase signalling. Br. J. Pharmacol. 174 (4), 287–301. 10.1111/bph.13688 27930802 PMC5289944

[B143] Khan M. A. Hashim M. J. Mustafa H. Baniyas M. Y. Al Suwaidi S. AlKatheeri R. (2020). Global epidemiology of ischemic heart disease: Results from the global burden of disease study. Cureus 12 (7), e9349. 10.7759/cureus.9349 32742886 PMC7384703

[B144] Khera A. V. Emdin C. A. Drake I. Natarajan P. Bick A. G. Cook N. R. (2016). Genetic risk, adherence to a healthy lifestyle, and coronary disease. N. Engl. J. Med. 375 (24), 2349–2358. 10.1056/NEJMoa1605086 27959714 PMC5338864

[B145] Kiebish M. A. Yang K. Sims H. F. Jenkins C. M. Liu X. Mancuso D. J. (2012). Myocardial regulation of lipidomic flux by cardiolipin synthase: Setting the beat for bioenergetic efficiency. J. Biol. Chem. 287 (30), 25086–25097. 10.1074/jbc.M112.340521 22584571 PMC3408154

[B146] Kirichok Y. Krapivinsky G. Clapham D. E. (2004). The mitochondrial calcium uniporter is a highly selective ion channel. Nature 427 (6972), 360–364. 10.1038/nature02246 14737170

[B147] Kitada T. Asakawa S. Hattori N. Matsumine H. Yamamura Y. Minoshima S. (1998). Mutations in the parkin gene cause autosomal recessive juvenile parkinsonism. Nature 392 (6676), 605–608. 10.1038/33416 9560156

[B148] Kloner R. A. Hale S. L. Dai W. Gorman R. C. Shuto T. Koomalsingh K. J. (2012). Reduction of ischemia/reperfusion injury with bendavia, a mitochondria-targeting cytoprotective Peptide. J. Am. Heart Assoc. 1 (3), e001644. 10.1161/JAHA.112.001644 23130143 PMC3487333

[B149] Klumpe I. Savvatis K. Westermann D. Tschope C. Rauch U. Landmesser U. (2016). Transgenic overexpression of adenine nucleotide translocase 1 protects ischemic hearts against oxidative stress. J. Mol. Med. 94 (6), 645–653. 10.1007/s00109-016-1413-4 27080394

[B150] Koyano F. Matsuda N. (2015). Molecular mechanisms underlying PINK1 and Parkin catalyzed ubiquitylation of substrates on damaged mitochondria. Biochim. Biophys. Acta 1853 (10), 2791–2796. 10.1016/j.bbamcr.2015.02.009 25700839

[B151] Koyano F. Okatsu K. Kosako H. Tamura Y. Go E. Kimura M. (2014). Ubiquitin is phosphorylated by PINK1 to activate parkin. Nature 510 (7503), 162–166. 10.1038/nature13392 24784582

[B152] Kubli D. A. Zhang X. Lee Y. Hanna R. A. Quinsay M. N. Nguyen C. K. (2013). Parkin protein deficiency exacerbates cardiac injury and reduces survival following myocardial infarction. J. Biol. Chem. 288 (2), 915–926. 10.1074/jbc.M112.411363 23152496 PMC3543040

[B153] Kupr B. Handschin C. (2015). Complex coordination of cell plasticity by a PGC-1α-controlled transcriptional network in skeletal muscle. Front. Physiol. 6, 325. 10.3389/fphys.2015.00325 26617528 PMC4639707

[B154] Lackner L. L. Ping H. Graef M. Murley A. Nunnari J. (2013). Endoplasmic reticulum-associated mitochondria-cortex tether functions in the distribution and inheritance of mitochondria. Proc. Natl. Acad. Sci. U. S. A. 110 (6), E458–E467. 10.1073/pnas.1215232110 23341591 PMC3568303

[B155] Lalloyer F. Staels B. (2010). Fibrates, glitazones, and peroxisome proliferator-activated receptors. Arterioscler. Thromb. Vasc. Biol. 30 (5), 894–899. 10.1161/ATVBAHA.108.179689 20393155 PMC2997800

[B156] Lawrence E. J. Mandato C. A. (2013). Mitochondria localize to the cleavage furrow in mammalian cytokinesis. PLoS One 8 (8), e72886. 10.1371/journal.pone.0072886 23991162 PMC3749163

[B157] Lee F. Y. Shao P. L. Wallace C. G. Chua S. Sung P. H. Ko S. F. (2018). Combined therapy with SS31 and mitochondria mitigates myocardial ischemia-reperfusion injury in rats. Int. J. Mol. Sci. 19 (9), 2782. 10.3390/ijms19092782 30223594 PMC6164143

[B158] Lee H. Yoon Y. (2016). Mitochondrial fission and fusion. Biochem. Soc. Trans. 44 (6), 1725–1735. 10.1042/BST20160129 27913683

[B159] Lee S. Min K. T. (2018). The interface between ER and mitochondria: Molecular compositions and functions. Mol. Cells 41 (12), 1000–1007. 10.14348/molcells.2018.0438 30590907 PMC6315321

[B160] Lee Y. Lee H. Y. Hanna R. A. Gustafsson A. B. (2011). Mitochondrial autophagy by Bnip3 involves Drp1-mediated mitochondrial fission and recruitment of Parkin in cardiac myocytes. Am. J. Physiol. Heart Circ. Physiol. 301 (5), H1924–H1931. 10.1152/ajpheart.00368.2011 21890690 PMC3213962

[B161] Lesnefsky E. J. Tandler B. Ye J. Slabe T. J. Turkaly J. Hoppel C. L. (1997). Myocardial ischemia decreases oxidative phosphorylation through cytochrome oxidase in subsarcolemmal mitochondria. Am. J. Physiol. 273 (3), H1544–H1554. 10.1152/ajpheart.1997.273.3.H1544 9321848

[B162] Lewis S. C. Uchiyama L. F. Nunnari J. (2016). ER-mitochondria contacts couple mtDNA synthesis with mitochondrial division in human cells. Science 353 (6296), aaf5549. 10.1126/science.aaf5549 27418514 PMC5554545

[B163] Li H. S. Zhou Y. N. Li L. Li S. F. Long D. Chen X. L. (2019a). HIF-1α protects against oxidative stress by directly targeting mitochondria. Redox Biol. 25, 101109. 10.1016/j.redox.2019.101109 30686776 PMC6859547

[B164] Li J. Dang X. Franco A. Dorn G. W. (2022a). 2ndReciprocal regulation of mitofusin 2-mediated mitophagy and mitochondrial fusion by different PINK1 phosphorylation events. Front. Cell Dev. Biol. 10, 868465. 10.3389/fcell.2022.868465 35646911 PMC9133611

[B165] Li J. Donath S. Li Y. Qin D. Prabhakar B. S. Li P. (2010). miR-30 regulates mitochondrial fission through targeting p53 and the dynamin-related protein-1 pathway. PLoS Genet. 6 (1), e1000795. 10.1371/journal.pgen.1000795 20062521 PMC2793031

[B166] Li X. Jia P. Huang Z. Liu S. Miao J. Guo Y. (2019b). Lycopene protects against myocardial ischemia-reperfusion injury by inhibiting mitochondrial permeability transition pore opening. Drug Des. devel. Ther. 13, 2331–2342. 10.2147/DDDT.S194753 PMC6635826 31371925

[B167] Li Y. Q. Jiao Y. Liu Y. N. Fu J. Y. Sun L. K. Su J. (2022b). PGC-1α protects from myocardial ischaemia-reperfusion injury by regulating mitonuclear communication. J. Cell. Mol. Med. 26 (3), 593–600. 10.1111/jcmm.16236 33470050 PMC8817131

[B169] Lin Y. Huang S. Chen Y. Wu Z. Liang Z. Zou M. (2020). Helix B surface peptide protects cardiomyocytes from hypoxia/reoxygenation-induced autophagy through the PI3K/akt pathway. J. Cardiovasc. Pharmacol. 76 (2), 181–188. 10.1097/FJC.0000000000000849 32404595

[B170] Lincoff A. M. Roe M. Aylward P. Galla J. Rynkiewicz A. Guetta V. (2014). Inhibition of delta-protein kinase C by delcasertib as an adjunct to primary percutaneous coronary intervention for acute anterior ST-segment elevation myocardial infarction: Results of the PROTECTION AMI randomized controlled trial. Eur. Heart J. 35 (37), 2516–2523. 10.1093/eurheartj/ehu177 24796339

[B171] Liu G. Zhang H. Hao F. Hao J. Pan L. Zhao Q. (2018). Clusterin reduces cold ischemia-reperfusion injury in heart transplantation through regulation of NF-kB signaling and bax/bcl-xL expression. Cell. Physiol. biochem. 45 (3), 1003–1012. 10.1159/000487295 29428944

[B172] Liu J. Wang L. N. (2017). Peroxisome proliferator-activated receptor gamma agonists for preventing recurrent stroke and other vascular events in people with stroke or transient ischaemic attack. Cochrane Database Syst. Rev. 12, CD010693. 10.1002/14651858.CD010693.pub4 29197071 PMC6486113

[B173] Liu L. Feng D. Chen G. Chen M. Zheng Q. Song P. (2012). Mitochondrial outer-membrane protein FUNDC1 mediates hypoxia-induced mitophagy in mammalian cells. Nat. Cell Biol. 14 (2), 177–185. 10.1038/ncb2422 22267086

[B174] Liu Y. J. McIntyre R. L. Janssens G. E. Houtkooper R. H. (2020). Mitochondrial fission and fusion: A dynamic role in aging and potential target for age-related disease. Mech. Ageing Dev. 186, 111212. 10.1016/j.mad.2020.111212 32017944

[B175] Liu Y. Zou J. Liu X. Zhang Q. (2019). MicroRNA-138 attenuates myocardial ischemia reperfusion injury through inhibiting mitochondria-mediated apoptosis by targeting HIF1-α. Exp. Ther. Med. 18 (5), 3325–3332. 10.3892/etm.2019.7976 31602205 PMC6777330

[B176] Lu F. H. Tian Z. Zhang W. H. Zhao Y. J. Li H. L. Ren H. (2010). Calcium-sensing receptors regulate cardiomyocyte Ca2+ signaling via the sarcoplasmic reticulum-mitochondrion interface during hypoxia/reoxygenation. J. Biomed. Sci. 17, 50. 10.1186/1423-0127-17-50 20565791 PMC2908572

[B177] Lu X. Thai P. N. Lu S. Pu J. Bers D. M. (2019). Intrafibrillar and perinuclear mitochondrial heterogeneity in adult cardiac myocytes. J. Mol. Cell. Cardiol. 136, 72–84. 10.1016/j.yjmcc.2019.08.013 31491377 PMC7173146

[B178] Madrid-Miller A. Moreno-Ruiz L. A. Borrayo-Sanchez G. Almeida-Gutierrez E. Martinez-Gomez D. F. Jauregui-Aguilar R. (2010). Ipact of bezafibrate treatment in patients with hyperfibrinogenemia and ST-elevation acute myocardial infarction: A randomized clinical trial. Cir. Cir. 78 (3), 229–237. 20642906

[B179] Majumder P. K. Pandey P. Sun X. Cheng K. Datta R. Saxena S. (2000). Mitochondrial translocation of protein kinase C delta in phorbol ester-induced cytochrome c release and apoptosis. J. Biol. Chem. 275 (29), 21793–21796. 10.1074/jbc.C000048200 10818086

[B180] Maneechote C. Palee S. Chattipakorn S. C. Chattipakorn N. (2017). Roles of mitochondrial dynamics modulators in cardiac ischaemia/reperfusion injury. J. Cell. Mol. Med. 21 (11), 2643–2653. 10.1111/jcmm.13330 28941171 PMC5661112

[B181] Maneechote C. Palee S. Kerdphoo S. Jaiwongkam T. Chattipakorn S. C. Chattipakorn N. (2019). Balancing mitochondrial dynamics via increasing mitochondrial fusion attenuates infarct size and left ventricular dysfunction in rats with cardiac ischemia/reperfusion injury. Clin. Sci. 133 (3), 497–513. 10.1042/CS20190014 30705107

[B182] Marchi S. Bittremieux M. Missiroli S. Morganti C. Patergnani S. Sbano L. (2017). Endoplasmic reticulum-mitochondria communication through Ca(2+) signaling: The importance of mitochondria-associated membranes (MAMs). Adv. Exp. Med. Biol. 997, 49–67. 10.1007/978-981-10-4567-7_4 28815521

[B183] Marchi S. Patergnani S. Pinton P. (2014). The endoplasmic reticulum-mitochondria connection: One touch, multiple functions. Biochim. Biophys. Acta 1837 (4), 461–469. 10.1016/j.bbabio.2013.10.015 24211533

[B184] Masuzawa A. Black K. M. Pacak C. A. Ericsson M. Barnett R. J. Drumm C. (2013). Transplantation of autologously derived mitochondria protects the heart from ischemia-reperfusion injury. Am. J. Physiol. Heart Circ. Physiol. 304 (7), H966–H982. 10.1152/ajpheart.00883.2012 23355340 PMC3625892

[B185] Mattie S. Riemer J. Wideman J. G. McBride H. M. (2018). A new mitofusin topology places the redox-regulated C terminus in the mitochondrial intermembrane space. J. Cell Biol. 217 (2), 507–515. 10.1083/jcb.201611194 29212658 PMC5800796

[B186] Mellbin L. G. Malmberg K. Norhammar A. Wedel H. Ryden L. Investigators D. (2008). The impact of glucose lowering treatment on long-term prognosis in patients with type 2 diabetes and myocardial infarction: A report from the DIGAMI 2 trial. Eur. Heart J. 29 (2), 166–176. 10.1093/eurheartj/ehm518 18156614

[B187] Missiroli S. Danese A. Iannitti T. Patergnani S. Perrone M. Previati M. (2017). Endoplasmic reticulum-mitochondria Ca(2+) crosstalk in the control of the tumor cell fate. Biochim. Biophys. Acta. Mol. Cell Res. 1864 (6), 858–864. 10.1016/j.bbamcr.2016.12.024 28064002

[B188] Miura T. Miki T. Yano T. (2010). Role of the gap junction in ischemic preconditioning in the heart. Am. J. Physiol. Heart Circ. Physiol. 298 (4), H1115–H1125. 10.1152/ajpheart.00879.2009 20118409

[B189] Montes de Oca Balderas P. (2021). Mitochondria-plasma membrane interactions and communication. J. Biol. Chem. 297 (4), 101164. 10.1016/j.jbc.2021.101164 34481840 PMC8503596

[B190] Morciano G. Giorgi C. Bonora M. Punzetti S. Pavasini R. Wieckowski M. R. (2015). Molecular identity of the mitochondrial permeability transition pore and its role in ischemia-reperfusion injury. J. Mol. Cell. Cardiol. 78, 142–153. 10.1016/j.yjmcc.2014.08.015 25172387

[B191] Morciano G. Patergnani S. Bonora M. Pedriali G. Tarocco A. Bouhamida E. (2020). Mitophagy in cardiovascular diseases. J. Clin. Med. 9 (3), 892. 10.3390/jcm9030892 32214047 PMC7141512

[B192] Morciano G. Patergnani S. Pedriali G. Cimaglia P. Mikus E. Calvi S. (2021a). Impairment of mitophagy and autophagy accompanies calcific aortic valve stenosis favouring cell death and the severity of disease. Cardiovasc. Res. 118, 2548–2559. 10.1093/cvr/cvab267 34375401

[B193] Morciano G. Pedriali G. Bonora M. Pavasini R. Mikus E. Calvi S. (2021b). A naturally occurring mutation in ATP synthase subunit c is associated with increased damage following hypoxia/reoxygenation in STEMI patients. Cell Rep. 35 (2), 108983. 10.1016/j.celrep.2021.108983 33852870

[B194] Morciano G. Pedriali G. Mikus E. Cimaglia P. Calvi S. Pavasini R. (2022). Similarities between fibroblasts and cardiomyocytes in the study of the permeability transition pore. Eur. J. Clin. Invest. 52, e13764. 10.1111/eci.13764 35289397 PMC9286368

[B195] Morciano G. Preti D. Pedriali G. Aquila G. Missiroli S. Fantinati A. (2018). Discovery of novel 1, 3, 8-triazaspiro[4.5]decane derivatives that target the c subunit of F1/FO-adenosine triphosphate (ATP) synthase for the treatment of reperfusion damage in myocardial infarction. J. Med. Chem. 61 (16), 7131–7143. 10.1021/acs.jmedchem.8b00278 30060655

[B196] Murphy E. Ardehali H. Balaban R. S. DiLisa F. Dorn G. W. 2nd Kitsis R. N. (2016). Mitochondrial function, biology, and role in disease: A scientific statement from the American heart association. Circ. Res. 118 (12), 1960–1991. 10.1161/RES.0000000000000104 27126807 PMC6398603

[B197] Murray C. J. Lopez A. D. (1997). Mortality by cause for eight regions of the world: Global burden of disease study. Lancet 349 (9061), 1269–1276. 10.1016/S0140-6736(96)07493-4 9142060

[B198] Nakagawa T. Shimizu S. Watanabe T. Yamaguchi O. Otsu K. Yamagata H. (2005). Cyclophilin D-dependent mitochondrial permeability transition regulates some necrotic but not apoptotic cell death. Nature 434 (7033), 652–658. 10.1038/nature03317 15800626

[B199] Neginskaya M. A. Solesio M. E. Berezhnaya E. V. Amodeo G. F. Mnatsakanyan N. Jonas E. A. (2019). ATP synthase C-Subunit-Deficient mitochondria have a small cyclosporine A-sensitive channel, but lack the permeability transition pore. Cell Rep. 26 (1), 11–17. 10.1016/j.celrep.2018.12.033 30605668 PMC6521848

[B200] Ng S. De Clercq I. Van Aken O. Law S. R. Ivanova A. Willems P. (2014). Anterograde and retrograde regulation of nuclear genes encoding mitochondrial proteins during growth, development, and stress. Mol. Plant 7 (7), 1075–1093. 10.1093/mp/ssu037 24711293

[B201] Nishihara M. Miura T. Miki T. Tanno M. Yano T. Naitoh K. (2007). Modulation of the mitochondrial permeability transition pore complex in GSK-3beta-mediated myocardial protection. J. Mol. Cell. Cardiol. 43 (5), 564–570. 10.1016/j.yjmcc.2007.08.010 17931653

[B202] Nissen S. E. Wolski K. (2007). Effect of rosiglitazone on the risk of myocardial infarction and death from cardiovascular causes. N. Engl. J. Med. 356 (24), 2457–2471. 10.1056/NEJMoa072761 17517853

[B203] O'Rourke B. Blatter L. A. (2009). Mitochondrial Ca2+ uptake: Tortoise or hare? J. Mol. Cell. Cardiol. 46 (6), 767–774. 10.1016/j.yjmcc.2008.12.011 19162034 PMC4005816

[B204] Ong S. B. Gustafsson A. B. (2012). New roles for mitochondria in cell death in the reperfused myocardium. Cardiovasc. Res. 94 (2), 190–196. 10.1093/cvr/cvr312 22108916 PMC3331612

[B205] Ong S. B. Kwek X. Y. Katwadi K. Hernandez-Resendiz S. Crespo-Avilan G. E. Ismail N. I. (2019). Targeting mitochondrial fission using mdivi-1 in A clinically relevant large animal model of acute myocardial infarction: A pilot study. Int. J. Mol. Sci. 20 (16), 3972. 10.3390/ijms20163972 31443187 PMC6720595

[B206] Ong S. B. Subrayan S. Lim S. Y. Yellon D. M. Davidson S. M. Hausenloy D. J. (2010). Inhibiting mitochondrial fission protects the heart against ischemia/reperfusion injury. Circulation 121 (18), 2012–2022. 10.1161/CIRCULATIONAHA.109.906610 20421521

[B207] Ong S. G. Hausenloy D. J. (2012). Hypoxia-inducible factor as a therapeutic target for cardioprotection. Pharmacol. Ther. 136 (1), 69–81. 10.1016/j.pharmthera.2012.07.005 22800800

[B208] Ong S. G. Lee W. H. Theodorou L. Kodo K. Lim S. Y. Shukla D. H. (2014). HIF-1 reduces ischaemia-reperfusion injury in the heart by targeting the mitochondrial permeability transition pore. Cardiovasc. Res. 104 (1), 24–36. 10.1093/cvr/cvu172 25063991

[B209] Ottani F. Latini R. Staszewsky L. La Vecchia L. Locuratolo N. Sicuro M. (2016). Cyclosporine A in reperfused myocardial infarction: The multicenter, controlled, open-label cycle trial. J. Am. Coll. Cardiol. 67 (4), 365–374. 10.1016/j.jacc.2015.10.081 26821623

[B210] Paillard M. Tubbs E. Thiebaut P. A. Gomez L. Fauconnier J. Da Silva C. C. (2013). Depressing mitochondria-reticulum interactions protects cardiomyocytes from lethal hypoxia-reoxygenation injury. Circulation 128 (14), 1555–1565. 10.1161/CIRCULATIONAHA.113.001225 23983249

[B211] Palee S. Higgins L. Leech T. Chattipakorn S. C. Chattipakorn N. (2020). Acute metformin treatment provides cardioprotection via improved mitochondrial function in cardiac ischemia/reperfusion injury. Biomed. Pharmacother. 130, 110604. 10.1016/j.biopha.2020.110604 32777704

[B212] Palee S. Weerateerangkul P. Surinkeaw S. Chattipakorn S. Chattipakorn N. (2011). Effect of rosiglitazone on cardiac electrophysiology, infarct size and mitochondrial function in ischaemia and reperfusion of swine and rat heart. Exp. Physiol. 96 (8), 778–789. 10.1113/expphysiol.2011.057885 21666037

[B213] Palikaras K. Lionaki E. Tavernarakis N. (2015). Coordination of mitophagy and mitochondrial biogenesis during ageing in *C. elegans* . Nature 521 (7553), 525–528. 10.1038/nature14300 25896323

[B214] Panel M. Ahmed-Belkacem A. Ruiz I. Guichou J. F. Pawlotsky J. M. Ghaleh B. (2021). A phenyl-pyrrolidine derivative reveals a dual inhibition mechanism of myocardial mitochondrial permeability transition pore, which is limited by its myocardial distribution. J. Pharmacol. Exp. Ther. 376 (3), 348–357. 10.1124/jpet.120.000359 33303698

[B215] Papanicolaou K. N. Khairallah R. J. Ngoh G. A. Chikando A. Luptak I. O'Shea K. M. (2011). Mitofusin-2 maintains mitochondrial structure and contributes to stress-induced permeability transition in cardiac myocytes. Mol. Cell. Biol. 31 (6), 1309–1328. 10.1128/MCB.00911-10 21245373 PMC3067905

[B216] Park M. K. Ashby M. C. Erdemli G. Petersen O. H. Tepikin A. V. (2001). Perinuclear, perigranular and sub-plasmalemmal mitochondria have distinct functions in the regulation of cellular calcium transport. EMBO J. 20 (8), 1863–1874. 10.1093/emboj/20.8.1863 11296220 PMC125431

[B217] Parodi-Rullan R. M. Soto-Prado J. Vega-Lugo J. Chapa-Dubocq X. Diaz-Cordero S. I. Javadov S. (2018). Divergent effects of cyclophilin-D inhibition on the female rat heart: Acute versus chronic post-myocardial infarction. Cell. Physiol. biochem. 50 (1), 288–303. 10.1159/000494006 30282073 PMC6247791

[B218] Patergnani S. Danese A. Bouhamida E. Aguiari G. Previati M. Pinton P. (2020). Various aspects of calcium signaling in the regulation of apoptosis, autophagy, cell proliferation, and cancer. Int. J. Mol. Sci. 21 (21), 8323. 10.3390/ijms21218323 33171939 PMC7664196

[B219] Pedriali G. Morciano G. Patergnani S. Cimaglia P. Morelli C. Mikus E. (2020). Aortic valve stenosis and mitochondrial dysfunctions: Clinical and molecular perspectives. Int. J. Mol. Sci. 21 (14), 4899. 10.3390/ijms21144899 32664529 PMC7402290

[B220] Pedriali G. Rimessi A. Sbano L. Giorgi C. Wieckowski M. R. Previati M. (2017). Regulation of endoplasmic reticulum-mitochondria Ca(2+) transfer and its importance for anti-cancer therapies. Front. Oncol. 7, 180. 10.3389/fonc.2017.00180 28913175 PMC5583168

[B221] Perrone M. Caroccia N. Genovese I. Missiroli S. Modesti L. Pedriali G. (2020). The role of mitochondria-associated membranes in cellular homeostasis and diseases. Int. Rev. Cell Mol. Biol. 350, 119–196. 10.1016/bs.ircmb.2019.11.002 32138899

[B222] Petrosillo G. Casanova G. Matera M. Ruggiero F. M. Paradies G. (2006). Interaction of peroxidized cardiolipin with rat-heart mitochondrial membranes: Induction of permeability transition and cytochrome c release. FEBS Lett. 580 (27), 6311–6316. 10.1016/j.febslet.2006.10.036 17083938

[B223] Petrosillo G. Colantuono G. Moro N. Ruggiero F. M. Tiravanti E. Di Venosa N. (2009). Melatonin protects against heart ischemia-reperfusion injury by inhibiting mitochondrial permeability transition pore opening. Am. J. Physiol. Heart Circ. Physiol. 297 (4), H1487–H1493. 10.1152/ajpheart.00163.2009 19684190

[B224] Ping H. A. Kraft L. M. Chen W. Nilles A. E. Lackner L. L. (2016). Num1 anchors mitochondria to the plasma membrane via two domains with different lipid binding specificities. J. Cell Biol. 213 (5), 513–524. 10.1083/jcb.201511021 27241910 PMC4896055

[B225] Pinton P. (2018). Mitochondria-associated membranes (MAMs) and pathologies. Cell Death Dis. 9 (4), 413. 10.1038/s41419-018-0424-1 29549303 PMC5856760

[B226] Piot C. Croisille P. Staat P. Thibault H. Rioufol G. Mewton N. (2008). Effect of cyclosporine on reperfusion injury in acute myocardial infarction. N. Engl. J. Med. 359 (5), 473–481. 10.1056/NEJMoa071142 18669426

[B227] Piper H. M. Meuter K. Schafer C. (2003). Cellular mechanisms of ischemia-reperfusion injury. Ann. Thorac. Surg. 75 (2), S644–S648. 10.1016/s0003-4975(02)04686-6 12607706

[B228] Pisano A. Cerbelli B. Perli E. Pelullo M. Bargelli V. Preziuso C. (2016). Impaired mitochondrial biogenesis is a common feature to myocardial hypertrophy and end-stage ischemic heart failure. Cardiovasc. Pathol. 25 (2), 103–112. 10.1016/j.carpath.2015.09.009 26764143 PMC4758811

[B229] Prime T. A. Blaikie F. H. Evans C. Nadtochiy S. M. James A. M. Dahm C. C. (2009). A mitochondria-targeted S-nitrosothiol modulates respiration, nitrosates thiols, and protects against ischemia-reperfusion injury. Proc. Natl. Acad. Sci. U. S. A. 106 (26), 10764–10769. 10.1073/pnas.0903250106 19528654 PMC2696550

[B230] Qi X. Wang J. (2020). Melatonin improves mitochondrial biogenesis through the AMPK/PGC1α pathway to attenuate ischemia/reperfusion-induced myocardial damage. Aging (Albany NY) 12 (8), 7299–7312. 10.18632/aging.103078 32305957 PMC7202489

[B231] Qiao X. Jia S. Ye J. Fang X. Zhang C. Cao Y. (2017). PTPIP51 regulates mouse cardiac ischemia/reperfusion through mediating the mitochondria-SR junction. Sci. Rep. 7, 45379. 10.1038/srep45379 28345618 PMC5366942

[B232] Ramaccini D. Montoya-Uribe V. Aan F. J. Modesti L. Potes Y. Wieckowski M. R. (2020). Mitochondrial function and dysfunction in dilated cardiomyopathy. Front. Cell Dev. Biol. 8, 624216. 10.3389/fcell.2020.624216 33511136 PMC7835522

[B233] Ramaccini D. Pedriali G. Perrone M. Bouhamida E. Modesti L. Wieckowski M. R. (2022). Some insights into the regulation of cardiac physiology and pathology by the Hippo pathway. Biomedicines 10 (3), 726. 10.3390/biomedicines10030726 35327528 PMC8945338

[B234] Ramesh V. Sharma V. K. Sheu S. S. Franzini-Armstrong C. (1998). Structural proximity of mitochondria to calcium release units in rat ventricular myocardium may suggest a role in Ca2+ sequestration. Ann. N. Y. Acad. Sci. 853, 341–344. 10.1111/j.1749-6632.1998.tb08295.x 10603975

[B235] Regula K. M. Ens K. Kirshenbaum L. A. (2002). Inducible expression of BNIP3 provokes mitochondrial defects and hypoxia-mediated cell death of ventricular myocytes. Circ. Res. 91 (3), 226–231. 10.1161/01.res.0000029232.42227.16 12169648

[B236] Riehle C. Wende A. R. Zaha V. G. Pires K. M. Wayment B. Olsen C. (2011). PGC-1β deficiency accelerates the transition to heart failure in pressure overload hypertrophy. Circ. Res. 109 (7), 783–793. 10.1161/CIRCRESAHA.111.243964 21799152 PMC3175248

[B237] Rocha A. G. Franco A. Krezel A. M. Rumsey J. M. Alberti J. M. Knight W. C. (2018). MFN2 agonists reverse mitochondrial defects in preclinical models of Charcot-Marie-Tooth disease type 2A. Science 360 (6386), 336–341. 10.1126/science.aao1785 29674596 PMC6109362

[B238] Rodriguez-Sinovas A. Garcia-Dorado D. Ruiz-Meana M. Soler-Soler J. (2004). Enhanced effect of gap junction uncouplers on macroscopic electrical properties of reperfused myocardium. J. Physiol. 559 (1), 245–257. 10.1113/jphysiol.2004.065144 15218064 PMC1665057

[B239] Roth G. A. Mensah G. A. Johnson C. O. Addolorato G. Ammirati E. Baddour L. M. (2020). Global burden of cardiovascular diseases and risk factors, 1990-2019: Update from the GBD 2019 study. J. Am. Coll. Cardiol. 76 (25), 2982–3021. 10.1016/j.jacc.2020.11.010 33309175 PMC7755038

[B240] Sala-Mercado J. A. Wider J. Undyala V. V. Jahania S. Yoo W. Mentzer R. M. Jr. (2010). Profound cardioprotection with chloramphenicol succinate in the swine model of myocardial ischemia-reperfusion injury. Circulation 122 (11), S179–S184. 10.1161/CIRCULATIONAHA.109.928242 20837911 PMC3355994

[B241] Salvadori M. Rosso G. Bertoni E. (2015). Update on ischemia-reperfusion injury in kidney transplantation: Pathogenesis and treatment. World J. Transpl. 5 (2), 52–67. 10.5500/wjt.v5.i2.52 PMC4478600 26131407

[B242] Scarpulla R. C. (2008). Nuclear control of respiratory chain expression by nuclear respiratory factors and PGC-1-related coactivator. Ann. N. Y. Acad. Sci. 1147, 321–334. 10.1196/annals.1427.006 19076454 PMC2853241

[B243] Schinzel A. C. Takeuchi O. Huang Z. Fisher J. K. Zhou Z. Rubens J. (2005). Cyclophilin D is a component of mitochondrial permeability transition and mediates neuronal cell death after focal cerebral ischemia. Proc. Natl. Acad. Sci. U. S. A. 102 (34), 12005–12010. 10.1073/pnas.0505294102 16103352 PMC1189333

[B244] Sciarretta S. Maejima Y. Zablocki D. Sadoshima J. (2018). The role of autophagy in the heart. Annu. Rev. Physiol. 80, 1–26. 10.1146/annurev-physiol-021317-121427 29068766

[B245] Sharma V. K. Ramesh V. Franzini-Armstrong C. Sheu S. S. (2000). Transport of Ca2+ from sarcoplasmic reticulum to mitochondria in rat ventricular myocytes. J. Bioenerg. Biomembr. 32 (1), 97–104. 10.1023/a:1005520714221 11768767

[B246] Sharp W. W. Fang Y. H. Han M. Zhang H. J. Hong Z. Banathy A. (2014). Dynamin-related protein 1 (Drp1)-mediated diastolic dysfunction in myocardial ischemia-reperfusion injury: Therapeutic benefits of Drp1 inhibition to reduce mitochondrial fission. FASEB J. 28 (1), 316–326. 10.1096/fj.12-226225 24076965 PMC3868827

[B247] Shen T. Zheng M. Cao C. Chen C. Tang J. Zhang W. (2007). Mitofusin-2 is a major determinant of oxidative stress-mediated heart muscle cell apoptosis. J. Biol. Chem. 282 (32), 23354–23361. 10.1074/jbc.M702657200 17562700

[B248] Shimada T. Horita K. Murakami M. Ogura R. (1984). Morphological studies of different mitochondrial populations in monkey myocardial cells. Cell Tissue Res. 238 (3), 577–582. 10.1007/BF00219874 6525619

[B249] Shires S. E. Gustafsson A. B. (2015). Mitophagy and heart failure. J. Mol. Med. 93 (3), 253–262. 10.1007/s00109-015-1254-6 25609139 PMC4334711

[B250] Siddall H. K. Yellon D. M. Ong S. B. Mukherjee U. A. Burke N. Hall A. R. (2013). Loss of PINK1 increases the heart's vulnerability to ischemia-reperfusion injury. PLoS One 8 (4), e62400. 10.1371/journal.pone.0062400 23638067 PMC3639249

[B251] Smith R. A. Murphy M. P. (2010). Animal and human studies with the mitochondria-targeted antioxidant MitoQ. Ann. N. Y. Acad. Sci. 1201, 96–103. 10.1111/j.1749-6632.2010.05627.x 20649545

[B252] Song M. Gong G. Burelle Y. Gustafsson A. B. Kitsis R. N. Matkovich S. J. (2015). Interdependence of parkin-mediated mitophagy and mitochondrial fission in adult mouse hearts. Circ. Res. 117 (4), 346–351. 10.1161/CIRCRESAHA.117.306859 26038571 PMC4522211

[B253] Su Q. Xu Y. Cai R. Dai R. Yang X. Liu Y. (2021). miR-146a inhibits mitochondrial dysfunction and myocardial infarction by targeting cyclophilin D. Mol. Ther. Nucleic Acids 23, 1258–1271. 10.1016/j.omtn.2021.01.034 33717647 PMC7907681

[B254] Suen D. F. Narendra D. P. Tanaka A. Manfredi G. Youle R. J. (2010). Parkin overexpression selects against a deleterious mtDNA mutation in heteroplasmic cybrid cells. Proc. Natl. Acad. Sci. U. S. A. 107 (26), 11835–11840. 10.1073/pnas.0914569107 20547844 PMC2900690

[B255] Sun C. K. Chang L. T. Sheu J. J. Wang C. Y. Youssef A. A. Wu C. J. (2007). Losartan preserves integrity of cardiac gap junctions and PGC-1 alpha gene expression and prevents cellular apoptosis in remote area of left ventricular myocardium following acute myocardial infarction. Int. Heart J. 48 (4), 533–546. 10.1536/ihj.48.533 17827825

[B256] Sun H. Han G. Z. Su C. Y. Dai S. F. (1990). Pharmacokinetics and distribution of isotetrandrine in rats. Yao Xue Xue Bao 25 (4), 241–246. 2281784

[B257] Sunaga H. Koitabashi N. Iso T. Matsui H. Obokata M. Kawakami R. (2019). Activation of cardiac AMPK-FGF21 feed-forward loop in acute myocardial infarction: Role of adrenergic overdrive and lipolysis byproducts. Sci. Rep. 9 (1), 11841. 10.1038/s41598-019-48356-1 31413360 PMC6694166

[B258] Susin S. A. Lorenzo H. K. Zamzami N. Marzo I. Snow B. E. Brothers G. M. (1999). Molecular characterization of mitochondrial apoptosis-inducing factor. Nature 397 (6718), 441–446. 10.1038/17135 9989411

[B259] Szeto H. H. (2014). First-in-class cardiolipin-protective compound as a therapeutic agent to restore mitochondrial bioenergetics. Br. J. Pharmacol. 171 (8), 2029–2050. 10.1111/bph.12461 24117165 PMC3976620

[B260] Szeto H. H. (2008). Mitochondria-targeted cytoprotective peptides for ischemia-reperfusion injury. Antioxid. Redox Signal. 10 (3), 601–619. 10.1089/ars.2007.1892 17999629

[B261] Tan D. X. Manchester L. C. Qin L. Reiter R. J. (2016). Melatonin: A mitochondrial targeting molecule involving mitochondrial protection and dynamics. Int. J. Mol. Sci. 17 (12), 2124. 10.3390/ijms17122124 27999288 PMC5187924

[B262] Thomas L. W. Ashcroft M. (2019). Exploring the molecular interface between hypoxia-inducible factor signalling and mitochondria. Cell. Mol. Life Sci. 76 (9), 1759–1777. 10.1007/s00018-019-03039-y 30767037 PMC6453877

[B263] Tilokani L. Nagashima S. Paupe V. Prudent J. (2018). Mitochondrial dynamics: Overview of molecular mechanisms. Essays Biochem. 62 (3), 341–360. 10.1042/EBC20170104 30030364 PMC6056715

[B264] Tombo N. Imam Aliagan A. D. Feng Y. Singh H. Bopassa J. C. (2020). Cardiac ischemia/reperfusion stress reduces inner mitochondrial membrane protein (mitofilin) levels during early reperfusion. Free Radic. Biol. Med. 158, 181–194. 10.1016/j.freeradbiomed.2020.06.039 32726689 PMC7484119

[B265] Tong H. Imahashi K. Steenbergen C. Murphy E. (2002). Phosphorylation of glycogen synthase kinase-3beta during preconditioning through a phosphatidylinositol-3-kinase-dependent pathway is cardioprotective. Circ. Res. 90 (4), 377–379. 10.1161/01.res.0000012567.95445.55 11884365

[B266] Tong M. Zablocki D. Sadoshima J. (2020). The role of Drp1 in mitophagy and cell death in the heart. J. Mol. Cell. Cardiol. 142, 138–145. 10.1016/j.yjmcc.2020.04.015 32302592 PMC7545744

[B267] Tsutsumi Y. M. Kawaraguchi Y. Horikawa Y. T. Niesman I. R. Kidd M. W. Chin-Lee B. (2010). Role of caveolin-3 and glucose transporter-4 in isoflurane-induced delayed cardiac protection. Anesthesiology 112 (5), 1136–1145. 10.1097/ALN.0b013e3181d3d624 20418694 PMC2860616

[B268] Viscomi C. Bottani E. Civiletto G. Cerutti R. Moggio M. Fagiolari G. (2011). *In vivo* correction of COX deficiency by activation of the AMPK/PGC-1α axis. Cell Metab. 14 (1), 80–90. 10.1016/j.cmet.2011.04.011 21723506 PMC3130927

[B269] Volkers M. Konstandin M. H. Doroudgar S. Toko H. Quijada P. Din S. (2013). Mechanistic target of rapamycin complex 2 protects the heart from ischemic damage. Circulation 128 (19), 2132–2144. 10.1161/CIRCULATIONAHA.113.003638 24008870 PMC4131547

[B270] Wacquier B. Combettes L. Dupont G. (2020). Dual dynamics of mitochondrial permeability transition pore opening. Sci. Rep. 10 (1), 3924. 10.1038/s41598-020-60177-1 32127570 PMC7054270

[B271] Wang J. X. Jiao J. Q. Li Q. Long B. Wang K. Liu J. P. (2011). miR-499 regulates mitochondrial dynamics by targeting calcineurin and dynamin-related protein-1. Nat. Med. 17 (1), 71–78. 10.1038/nm.2282 21186368

[B272] Westermann B. (2015). The mitochondria-plasma membrane contact site. Curr. Opin. Cell Biol. 35, 1–6. 10.1016/j.ceb.2015.03.001 25801776

[B273] Whayne T. F. Jr. Saha S. P. (2019). Genetic risk, adherence to a healthy lifestyle, and ischemic heart disease. Curr. Cardiol. Rep. 21 (1), 1. 10.1007/s11886-019-1086-z 30631962

[B274] Wu D. Dasgupta A. Chen K. H. Neuber-Hess M. Patel J. Hurst T. E. (2020). Identification of novel dynamin-related protein 1 (Drp1) GTPase inhibitors: Therapeutic potential of Drpitor1 and Drpitor1a in cancer and cardiac ischemia-reperfusion injury. FASEB J. 34 (1), 1447–1464. 10.1096/fj.201901467R 31914641

[B275] Wu J. W. Hu H. Hua J. S. Ma L. K. (2022). ATPase inhibitory factor 1 protects the heart from acute myocardial ischemia/reperfusion injury through activating AMPK signaling pathway. Int. J. Biol. Sci. 18 (2), 731–741. 10.7150/ijbs.64956 35002521 PMC8741848

[B276] Wu S. Lu Q. Wang Q. Ding Y. Ma Z. Mao X. (2017). Binding of FUN14 domain containing 1 with inositol 1, 4, 5-trisphosphate receptor in mitochondria-associated endoplasmic reticulum membranes maintains mitochondrial dynamics and function in hearts *in vivo* . Circulation 136 (23), 2248–2266. 10.1161/CIRCULATIONAHA.117.030235 28942427 PMC5716911

[B277] Wu W. Lin C. Wu K. Jiang L. Wang X. Li W. (2016). FUNDC1 regulates mitochondrial dynamics at the ER-mitochondrial contact site under hypoxic conditions. EMBO J. 35 (13), 1368–1384. 10.15252/embj.201593102 27145933 PMC4864280

[B278] Xie J. R. Yu L. N. (2007). Cardioprotective effects of cyclosporine A in an *in vivo* model of myocardial ischemia and reperfusion. Acta Anaesthesiol. Scand. 51 (7), 909–913. 10.1111/j.1399-6576.2007.01342.x 17578461

[B279] Xin T. Lu C. (2020). Irisin activates Opa1-induced mitophagy to protect cardiomyocytes against apoptosis following myocardial infarction. Aging (Albany NY) 12 (5), 4474–4488. 10.18632/aging.102899 32155590 PMC7093202

[B280] Xu A. Szczepanek K. Hu Y. Lesnefsky E. J. Chen Q. (2013). Cardioprotection by modulation of mitochondrial respiration during ischemia-reperfusion: Role of apoptosis-inducing factor. Biochem. Biophys. Res. Commun. 435 (4), 627–633. 10.1016/j.bbrc.2013.05.033 23685150

[B281] Xu J. Ji J. Yan X. H. (2012). Cross-talk between AMPK and mTOR in regulating energy balance. Crit. Rev. Food Sci. Nutr. 52 (5), 373–381. 10.1080/10408398.2010.500245 22369257

[B282] Yan K. An T. Zhai M. Huang Y. Wang Q. Wang Y. (2019). Mitochondrial miR-762 regulates apoptosis and myocardial infarction by impairing ND2. Cell Death Dis. 10 (7), 500. 10.1038/s41419-019-1734-7 31235686 PMC6591419

[B283] Yang J. Chen L. Yang J. Ding J. Li S. Wu H. (2014). MicroRNA-22 targeting CBP protects against myocardial ischemia-reperfusion injury through anti-apoptosis in rats. Mol. Biol. Rep. 41 (1), 555–561. 10.1007/s11033-013-2891-x 24338162

[B284] Yu J. Maimaitili Y. Xie P. Wu J. J. Wang J. Yang Y. N. (2017). High glucose concentration abrogates sevoflurane post-conditioning cardioprotection by advancing mitochondrial fission but dynamin-related protein 1 inhibitor restores these effects. Acta Physiol. 220 (1), 83–98. 10.1111/apha.12812 27684054

[B285] Yue Tl T. L. Chen J. Bao W. Narayanan P. K. Bril A. Jiang W. (2001). *In vivo* myocardial protection from ischemia/reperfusion injury by the peroxisome proliferator-activated receptor-gamma agonist rosiglitazone. Circulation 104 (21), 2588–2594. 10.1161/hc4601.099403 11714655

[B286] Zhang C. X. Cheng Y. Liu D. Z. Liu M. Cui H. Zhang B. L. (2019a). Mitochondria-targeted cyclosporin A delivery system to treat myocardial ischemia reperfusion injury of rats. J. Nanobiotechnology 17 (1), 18. 10.1186/s12951-019-0451-9 30683110 PMC6346555

[B287] Zhang H. Bosch-Marce M. Shimoda L. A. Tan Y. S. Baek J. H. Wesley J. B. (2008). Mitochondrial autophagy is an HIF-1-dependent adaptive metabolic response to hypoxia. J. Biol. Chem. 283 (16), 10892–10903. 10.1074/jbc.M800102200 18281291 PMC2447655

[B288] Zhang J. Ney P. A. (2011). Mechanisms and biology of B-cell leukemia/lymphoma 2/adenovirus E1B interacting protein 3 and Nip-like protein X. Antioxid. Redox Signal. 14 (10), 1959–1969. 10.1089/ars.2010.3772 21126215 PMC3078493

[B289] Zhang J. Ney P. A. (2009). Role of BNIP3 and NIX in cell death, autophagy, and mitophagy. Cell Death Differ. 16 (7), 939–946. 10.1038/cdd.2009.16 19229244 PMC2768230

[B290] Zhang P. Guan P. Ye X. Lu Y. Hang Y. Su Y. (2022). SOCS6 promotes mitochondrial fission and cardiomyocyte apoptosis and is negatively regulated by quaking-mediated miR-19b. Oxid. Med. Cell. Longev. 2022, 1121323. 10.1155/2022/1121323 35126805 PMC8813278

[B291] Zhang Y. Liu D. Hu H. Zhang P. Xie R. Cui W. (2019b). HIF-1α/BNIP3 signaling pathway-induced-autophagy plays protective role during myocardial ischemia-reperfusion injury. Biomed. Pharmacother. 120, 109464. 10.1016/j.biopha.2019.109464 31590128

[B292] Zhang Y. Wang Y. Xu J. Tian F. Hu S. Chen Y. (2019c). Melatonin attenuates myocardial ischemia-reperfusion injury via improving mitochondrial fusion/mitophagy and activating the AMPK-OPA1 signaling pathways. J. Pineal Res. 66 (2), e12542. 10.1111/jpi.12542 30516280

[B293] Zhang Z. W. Xu X. C. Liu T. Yuan S. (2016). Mitochondrion-permeable antioxidants to treat ROS-burst-mediated acute diseases. Oxid. Med. Cell. Longev. 2016, 6859523. 10.1155/2016/6859523 26649144 PMC4663357

[B294] Zhao K. Zhao G. M. Wu D. Soong Y. Birk A. V. Schiller P. W. (2004). Cell-permeable peptide antioxidants targeted to inner mitochondrial membrane inhibit mitochondrial swelling, oxidative cell death, and reperfusion injury. J. Biol. Chem. 279 (33), 34682–34690. 10.1074/jbc.M402999200 15178689

[B295] Zhou H. Hu S. Jin Q. Shi C. Zhang Y. Zhu P. (2017). Mff-dependent mitochondrial fission contributes to the pathogenesis of cardiac microvasculature ischemia/reperfusion injury via induction of mROS-mediated cardiolipin oxidation and HK2/VDAC1 disassociation-involved mPTP opening. J. Am. Heart Assoc. 6 (3), e005328. 10.1161/JAHA.116.005328 28288978 PMC5524036

[B296] Zhou H. Wang J. Zhu P. Zhu H. Toan S. Hu S. (2018a). NR4A1 aggravates the cardiac microvascular ischemia reperfusion injury through suppressing FUNDC1-mediated mitophagy and promoting Mff-required mitochondrial fission by CK2α. Basic Res. Cardiol. 113 (4), 23. 10.1007/s00395-018-0682-1 29744594

[B297] Zhou H. Zhu P. Wang J. Zhu H. Ren J. Chen Y. (2018b). Pathogenesis of cardiac ischemia reperfusion injury is associated with CK2α-disturbed mitochondrial homeostasis via suppression of FUNDC1-related mitophagy. Cell Death Differ. 25 (6), 1080–1093. 10.1038/s41418-018-0086-7 29540794 PMC5988750

[B298] Zhu Z. D. Ye J. Y. Niu H. Ma Y. M. Fu X. M. Xia Z. H. (2018). Effects of microRNA-292-5p on myocardial ischemia-reperfusion injury through the peroxisome proliferator-activated receptor-α/-γ signaling pathway. Gene Ther. 25 (3), 234–248. 10.1038/s41434-018-0014-y 29670247

